# A single CAA interrupt in a DNA three-way junction containing a CAG repeat hairpin results in parity-dependent trapping

**DOI:** 10.1093/nar/gkae644

**Published:** 2024-07-23

**Authors:** Gillian M Cadden, Svea J Wilken, Steven W Magennis

**Affiliations:** School of Chemistry, University of Glasgow, Joseph Black Building, University Avenue, Glasgow G12 8QQ, UK; School of Chemistry, University of Glasgow, Joseph Black Building, University Avenue, Glasgow G12 8QQ, UK; School of Chemistry, University of Glasgow, Joseph Black Building, University Avenue, Glasgow G12 8QQ, UK

## Abstract

An increasing number of human disorders are attributed to genomic expansions of short tandem repeats (STRs). Secondary DNA structures formed by STRs are believed to play an important role in expansion, while the presence of nucleotide interruptions within the pure repeat sequence is known to delay the onset and progression of disease. We have used two single-molecule fluorescence techniques to analyse the structure and dynamics of DNA three-way junctions (3WJs) containing CAG repeat hairpin slipouts, with and without a single CAA interrupt. For a 3WJ with a (CAG)_10_ slipout, the CAA interrupt is preferentially located in the hairpin loop, and the branch migration dynamics are 4-fold slower than for the 3WJ with a pure (CAG)_10_, and 3-fold slower than a 3WJ with a pure (CAG)_40_ repeat. The (CAG)_11_ 3WJ with CAA interrupt adopts a conformation that places the interrupt in or near the hairpin loop, with similar dynamics to the pure (CAG)_10_ and (CAG)_11_ 3WJs. We have shown that changing a single nucleotide (G to A) in a pure repeat can have a large impact on 3WJ structure and dynamics, which may be important for the protective role of interrupts in repeat expansion diseases.

## Graphical Abstract

**Graphical Abstract:**
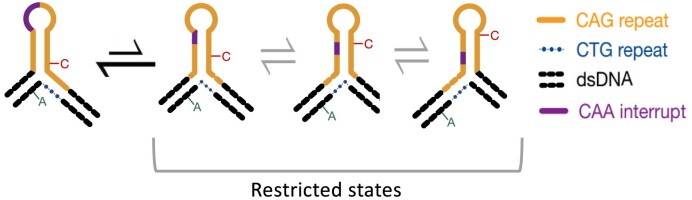

## Introduction

Approximately half of the human genome contains repeat sequences, spread across coding and non-coding regions (1). The ‘repeatome’ comprises 8% of the genomic DNA (2). Repeats have important roles in native functioning (3), including epigenetic modification, gene expression, in telomeres, and in the downstream regulation of RNA and proteins (4). Short tandem repeats (STRs), also known as microsatellites, are one major class of repeat, and are estimated to comprise 1–3% of the human genome (5). STRs are also central to a growing number (currently over 60) of severe monogenic human disorders referred to as repeat expansion diseases (REDs) (6,7). Recent genome-wide association studies (GWAS) have also implicated STRs in a range of polygenic diseases such as autism (8), amyotrophic lateral sclerosis (9), cancer (10) and schizophrenia (11).

For the expandable repeat sequences involved in REDs, the healthy range of repeats is relatively narrow, below around 40 repeats (6). Following expansion, the repeat number can increase by just a few repeats or by hundreds or thousands, depending on the RED. Furthermore, the length of the pure repeat sequence is inversely correlated with the age-of-onset, while interruptions in the repeat sequence are protective. In Huntington's disease, for example, where expanded CAG repeats leads to mutant Huntingtin protein containing extended polyglutamine tracts, it has been shown that loss of a CAA interruption can result in an age of clinical onset that is decades earlier, even though both CAG and CAA code for glutamine (12).

The majority of REDs concern STRs of 3–6 nucleotides. The most widely studied REDs involve trinucleotide repeats. REDs with repeats of CAG in an exon are the most common and are responsible for Huntington's disease (HD), several of the spinocerebellar ataxias (SCA1, 2, 3, 6, 7 and 17), and spinal and bulbar muscular atrophy (SBMA). A key feature of repeat DNA and RNA is their ability to form secondary structures such as hairpins, triplexes and G-quadruplexes (13). For CAG repeat disorders, both the CAG strand and the complementary CTG strand can form intramolecular hairpins as a result of their partial self-complementarity, with each triplet able to form two GC pairs with an A–A or T–T mismatch for CAG and CTG repeats, respectively (14–21). Such non-canonical structures have been proposed as key intermediates in most models of repeat expansion. While replication-related expansion can occur (22,23), growing evidence now supports a major role for replication-independent somatic instability in REDs (24). Furthermore, there is now a wealth of evidence that the key driver for somatic instability is non-canonical DNA repair, with the secondary structures as putative targets (6).

In an effort to understand the role of secondary structures on the onset and progression of REDs, a number of groups have recently used single-molecule Förster resonance energy transfer (smFRET), which is ideally suited to probing the structure and dynamics of biomolecules. The first reported studies were of hairpins containing repeats of TGGAA (25) where it was shown that slippage by one repeat unit could occur, depending on the parity of the repeat sequence; a subsequent study with CTG and CAG repeats also showed strand slippage by one repeat (26). Slippage was proposed to arise from local instability in the hairpin stem region, which was propagated along the hairpin.

Slipping by up to 2 CAGs was subsequently observed in CAG repeat hairpins, again with a sensitivity to odd-even parity (27). It was shown that 5′-AGCA-3′ tetraloops are more stable than 5′-CAG-3′ triloops, causing structures with even-numbered repeats to be less dynamic. This agreed with earlier ensemble work showing that hairpin structure and stability depended on repeat parity (28). It was also shown that placing a CAA interrupt near the hairpin loop stabilized the hairpin by avoiding a CA mismatch, and thereby suppressed dynamics (27). Studies of other repeat sequences and interrupts highlighted the fine balance of loop and stem energies in determining the preferred conformation (29). In support of this idea, slipping was found to be absent in CAG repeat hairpins with a poly-A loop (30). More recently, the role of one or two AGG interrupts in CGG repeat hairpins was investigated (31) building on earlier ensemble studies (32); observed changes in conformations and dynamics were again explained in terms of the energetics of different loop and stem configurations (31).

Related to these hairpin studies, we recently reported smFRET studies of DNA three-way junctions (3WJs) where one arm is a slip-out of trinucleotide repeats (CAG or CTG) of varying length (33,34), building on earlier work with non-repeat 3WJs (35,36). Whenever cell processing generates single-stranded DNA (ssDNA) containing complementary CAG and CTG repeats, there is an opportunity for ssDNA to self-hybridize and form two 3WJs through out-of-register annealing (6,37,38). We found that 3WJs where the triplet repeat sequence extends into the adjacent duplex can undergo two distinct types of re-arrangement: a localized conformational change at the branchpoint and a longer-range interconversion due to branch migration. Although such migration had been postulated (39), it was generally believed that such stable slip-outs were unable to undergo branch migration. In contrast, the dynamical nature of smaller repeat loops had been proposed previously, based on several ensemble fluorescence and thermodynamic studies (40–42).

**Figure 1.**
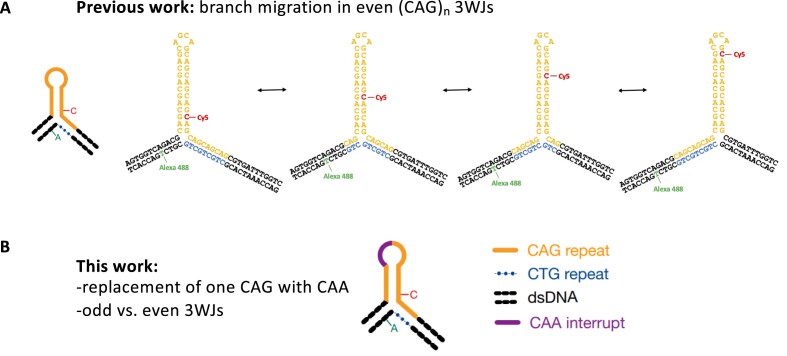
Dynamics of 3WJs containing a (CAG)_10_ or (CAG)_11_ hairpin in the presence and absence of a CAA interrupt. (**A**) 3WJs with an even number of CAG repeats in a hairpin slipout and with repeats extended into the adjacent duplex were previously shown to exhibit branchpoint migration. Four stable positional isomers are shown here for a (CAG)_10_ slipout. By labelling the 3WJs with a FRET pair, the structure and dynamics can be followed by smFRET. The cartoon on the left represents a 3WJ with a pure CAG repeat. (**B**) In this work, odd and even 3WJs, with and without a CAA interrupt, were studied.

In this work, we use two different single-molecule microscopy approaches (confocal and TIRF, for freely diffusing and immobilized molecules, respectively) to probe the effect of sequence interruption and repeat parity on the structure and dynamics of a 3WJ (Figure 1). Although interrupts in free hairpins have been shown to influence the slippage dynamics (27,29,31), the hairpins found in slipped-strand DNA must be considered in the context of a 3WJ. In free hairpins, the ends of the DNA are free to form overhangs, whereas in a 3WJ these ends are constrained by the branchpoint. Whether or not a single nucleotide interrupt could influence 3WJ branch structure and dynamics, especially given the known ion-induced stability of branched DNA, was an open question. We find that replacement of a single nucleotide (CAG to CAA) results in the preferential adoption of conformations in which the interrupt is in or near the hairpin loop, and with parity-dependent branch migration kinetics. For the even repeat, the single interrupt causes a 4-fold decrease in the rate of branch migration, and a 3-fold decrease relative to an uninterrupted 3WJ with 30 additional CAG repeats. This interplay of structure and dynamics may play an important role in the expansion of DNA repeats.

## Materials and methods

### Sample preparation

DNA oligonucleotides were synthesized and labelled by ATDBio (Oxford) using NHS-esters of Alexa 488 or Cy5. All 3WJs were annealed using two oligonucleotides at 5 μM (see Supplementary Data for sequences) in a buffer containing 10 mM Tris (pH 7.5) and 100 mM NaCl (20 ml). Buffers were prepared with Tris (Sigma-Aldrich), Tris-HCl (Sigma-Aldrich) and NaCl in ultrapure water (Direct Q3, Merck Millipore). The samples were heated for 5 minutes in a water bath at 90°C and allowed to anneal slowly overnight. Buffers for use in single-molecule experiments were pre-prepared and syringe-filtered (0.20 μm syringe filter (Millex, Millipore)) to ensure high purity.

For TIRF microscopy experiments, an imaging buffer composed of 20 mM Tris–HCl (pH 7.5), 6% w/v glucose (d-(+) glucose, Sigma Aldrich), 15 mM NaCl and 1 mM MgCl_2_ was used to prepare solutions of DNA to be immobilized onto the functionalized chamber surface and also as a wash buffer immediately prior to imaging. 3WJs were diluted to ∼20–50 pM DNA concentration and immobilised. 2 mg/ml glucose oxidase, 0.08 mg/mL glucose catalase (Sigma Aldrich)), and 2 mM Trolox ((+)-6-hydroxy-2,5,7,8-tetramethylchromane-2-carboxylic acid (Sigma Aldrich)) were added prior to imaging to reduce the rate of blinking and photobleaching of the dyes.

For MFD measurements, the buffer contained 20 mM Tris–HCl, 15 mM NaCl (pH 7.5), 1 mM MgCl_2_ and were cleaned using activated charcoal. Before diluting the DNA samples to the single-molecule level, vitamin C was added into the buffer (1 mM). Samples were typically diluted to ∼4 pM before measuring at room temperature (21 $ \pm$ 1°C).

### Confocal single-molecule measurements

MFD measurements were performed using a home-built system. The fluorescent donor molecule (Alexa 488) was excited by a linearly polarized laser (480 nm, 40 MHz, ∼60 ps FWHM; Picoquant, Germany). The laser light was focused into the dilute solution of labelled molecules by a water immersion objective (UPLAPO 60×, NA = 1.2, Olympus, UK). The average laser power at the sample was ca. 180 μW. All measurements were recorded at 21 ± 1°C. The data analysis used software written by the group of Prof. Claus Seidel (Heinrich Heine Universität, Düsseldorf). FRET efficiencies were measured from raw green and red signals and corrected for background and crosstalk.

### TIRF

Objective-type TIRF was performed using excitation with a 488 nm Stradus (Laser 2000, UK) diode laser, with flow cells placed on top of an inverted microscope (IX71, Olympus). The laser power at the sample during excitation was 30–35 μW. Fluorescence emission from a sample or bead slide was collected through the same 100×, NA = 1.49, oil immersion objective lens (Olympus) used for excitation and separated from scattered excitation via a 500 nm longpass filter and a 500 nm dichroic mirror (Chroma Technology Corp.). The fluorescence from sample molecules was collimated and split into donor and acceptor wavelengths (DV2 Multichannel Imaging System, Photometrics with a 565 LPXR dichroic, and 535/50 and 700/75 bandpass filters) and simultaneously imaged onto a cooled EMCCD camera (Evolve, Photometrics). This provided two separate, spatially identical images displaying donor and acceptor fluorescence.

All experiments in this work were carried out at room temperature (21 ± 1°C).

#### Recording movies

3WJs were immobilized on the cover slip surface using biotin-neutravidin interactions at an approximate concentration of 10 pM, such that approximately 200 spots were visible within the field of view. The fluorescence intensities of the emission from the Alexa488 (donor) and Cy5 (acceptor) dyes were detected using an EMCCD camera and the .tif movies were recorded by ImagePro-Plus 7.0 software using an exposure time of 50 ms.

#### Processing movies

Movies recorded as .tif files were processed using TwoTone 3.1. Images of bead slides, which emitted strong fluorescence in both donor and acceptor channels, were taken at the beginning of every TIRF experiment. These images were used to generate a mapping file in TwoTone to calibrate the donor and acceptor channels such that a spot observed in both channels is recognized as belonging to fluorescence from the same molecule. An intensity threshold can be set for each movie in both channels to ensure that only the brightest spots were analysed. Donor and acceptor intensities from individual molecules were recorded, for every frame of the movie, as a MATLAB array. Fluorescence vs. time trajectories were then visualized and selected using MATLAB procedures written in-house.

#### Transition analysis using MASH-FRET

All dynamic trajectories were analyzed individually without stitching using software packages ‘HaMMy’ (43) and ‘MASH-FRET’version 1.3.2 (44), in a similar method to that described previously by Bianco *et al.* (34). Unstitched time trajectories were used to produce transition density plots (TDPs) which were analyzed by fitting to K(K − 1) isotropic 2D Gaussians, where K is the number of FRET states. FRET values were derived from the Gaussian means and the associated errors from the average Gaussian sample standard deviations in MASH-FRET. The value of *K*_opt_ was inferred via ML-BIC optimization of models comprising K = 1 to 10 states with 30 initializations. Transition rate coefficients were estimated using the ‘state lifetimes’ method described by Hadzic *et al.* (44). Cumulative dwell time distributions were fit to single and bi-exponentials. To determine the rate coefficient of each transition, state lifetimes were multiplied by the weight of each corresponding cluster determined from the TDP analysis. An average value of the dwell time (τ_av_) was calculated by weighting each dwell time component (*τ*) by the amplitudes (A): τ_av_ = A_1_*τ*_1_ + A_2_*τ*_2_. The error in dwell time (3 × standard deviation) for each transition was determined using bootstrapped exponential fitting (BOBA-FRET) (45). To determine the error in the average dwell time, we separately calculated the error in A_1_*τ*_1_ (M) and A_2_*τ*_2_ (N) using $\Delta {\mathrm{M = }}( {{{{\mathrm{A}}}_1}{{\tau }_1}} ) \times \sqrt {{{{( {\frac{{\Delta {{{\mathrm{A}}}_{\mathrm{1}}}}}{{{{{\mathrm{A}}}_{\mathrm{1}}}}}} )}}^{\mathrm{2}}}{\mathrm{ + \ }}{{{( {\frac{{\Delta {{{\mathrm{\tau }}}_{\mathrm{1}}}}}{{{{{\mathrm{\tau }}}_{\mathrm{1}}}}}} )}}^{\mathrm{2}}}}$ and $\Delta {\mathrm{N = }}( {{{{\mathrm{A}}}_2}{{\tau }_2}} ) \times \sqrt {{{{( {\frac{{\Delta {{{\mathrm{A}}}_{\mathrm{2}}}}}{{{{{\mathrm{A}}}_{\mathrm{2}}}}}} )}}^{\mathrm{2}}}{\mathrm{ + \ }}{{{( {\frac{{\Delta {{{\mathrm{\tau }}}_{\mathrm{2}}}}}{{{{{\mathrm{\tau }}}_{\mathrm{2}}}}}} )}}^{\mathrm{2}}}}$ where ΔA and Δτ are the error in amplitude and dwell time errors from BOBA-FRET analysis. The overall error in τ_av_ (Δτ_av_) was calculated as $\Delta {{\tau }_{av}} = \sqrt {\Delta {{{\mathrm{M}}}^{2{\mathrm{\ }}}}{\mathrm{ + \ }}\Delta {{{\mathrm{N}}}^{\mathrm{2}}}}$.

## Results

We previously studied two types of 3WJ containing CAG repeat hairpin slipouts, formed by the annealing of two strands of DNA (33,34). One type, which we referred to as static 3WJs, only had (CAG)_10_ repeats in the hairpin, with flanking duplex regions of random sequence. In the other type, which we called mobile 3WJs, three CAG repeats extended from the hairpin (containing either 4, 6, 8, 10, 20, 30 or 40 repeats) into the duplex (Figure 1A). In this work, we study mobile 3WJs in which a single CAA replaces one of the CAG repeats in a hairpin composed of either (CAG)_10_ or (CAG)_11_; we also study a mobile 3WJ with a pure (CAG)_11_ hairpin (Figure 1B). Each 3WJ is labelled with two fluorescent dyes, one on each strand, allowing us to perform smFRET. All DNA sequences and dye positions are listed in the Supplementary Data. All samples were studied in a buffer containing 1mM MgCl_2_; we note that previous work had shown a weak dependence on MgCl_2_ concentration (33). We use two complementary FRET methods: total internal reflection fluorescence (TIRF) microscopy (46) for observing immobilized molecules, and multiparameter fluorescence detection (MFD) (47), which is a confocal method to study freely diffusing molecules.

### Single-molecule spectroscopy of (CAG)_10_ 3WJs containing a CAA interrupt

**Figure 2.**
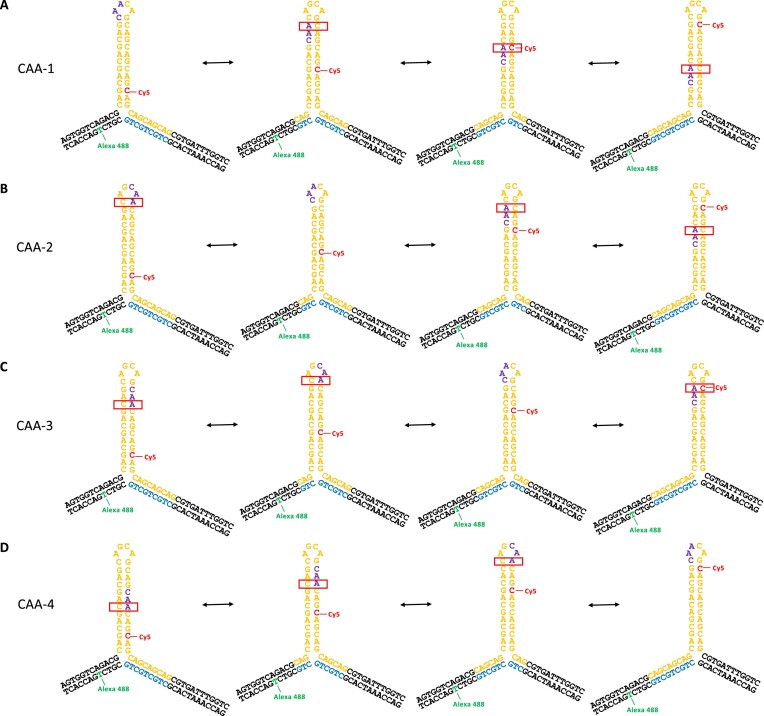
Possible stable positional isomers following branch migration for a (CAG)_10_ hairpin with one CAG replaced by CAA. The CAA interrupt is in one of four locations. (**A**) (CAG)_4_CAA(CAG)_5_ (‘CAA-1’). (**B**) (CAG)_5_CAA(CAG)_4_ (‘CAA-2’).(**C**) (CAG)_6_CAA(CAG)_3_ (‘CAA-3’). (**D**) (CAG)_7_CAA(CAG)_2_ (‘CAA-4’). CA mismatches for the positional isomers are indicated in a red box.

We showed previously that a mobile 3WJ with even numbers of either CAG or CTG repeats in the hairpin was able to undergo branch migration, whereby the point of extrusion of the hairpin from the duplex DNA shifts by up to 4 repeat units (e.g. see Figure 1A for a hairpin of 10 CAG repeats). For a pure repeat, each of these positional isomers is almost equivalent thermodynamically because they all have the same identity and number of canonical basepairs; this is in contrast to slippage in a free CAG hairpin, where slipping results in unpaired triplet overhangs. When an interrupt is added to the 3WJs, in this case switching G for A, then any structure where the CAA is located in the stem will have a CA mismatch in place of a CG basepair (Figure 2). We designed four 3WJs with a single CAA interrupt (Figure 2). These are identical to the pure repeat structure in Figure 1A, but with the replacement of either the fourth, fifth, sixth or seventh CAG by CAA (named CAA 1–4, respectively). For each of the four interrupt positions, there is a unique positional isomer in which there is no mismatch in the stem because the interrupt is located in the hairpin loop (Figure 2).

#### Confocal microscopy of freely diffusing (CAG)_10_ 3WJs with interrupts

Confocal MFD is used here to measure FRET for freely diffusing single 3WJs, revealing the FRET efficiency and donor fluorescence anisotropy for each molecule as a function of the donor fluorescence lifetime. The 2D histograms provide unambiguous evidence of FRET-related changes in the donor and acceptor fluorescence, and can reveal sub-populations of FRET states (47). FRET states that are stable on the timescale of diffusion through the confocal volume (few ms) lie on theoretical FRET lines in 2D plots of FRET efficiency or donor anisotropy vs donor lifetime. All of the 3WJ FRET states in this work lie on these lines, indicating the absence of sub-millisecond dynamics. Therefore, we have focused here on the 1D plot of FRET efficiency vs donor lifetime with full 2D MFD plots shown in the Supplementary Data.

**Figure 3.**
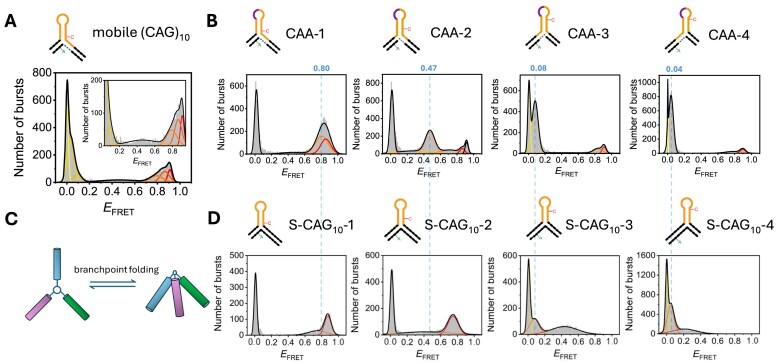
Confocal smFRET of freely diffusing (CAG)_10_ 3WJs with interrupts at different positions. (**A**) E_FRET_ histogram from MFD analysis of mobile (CAG)_10_ 3WJs without interrupts. (**B**) *E*_FRET_ histograms of mobile (CAG)_10_ 3WJs with CAA interrupts at positions 1–4 (see Supplementary Figure S1 for the full MFD plots). (**C**) Conformational flexibility at the branchpoint results in multiple FRET states for each positional isomer. (**D**) *E*_FRET_ histogram of static 3WJs (S-CAG_10_-1, S-CAG_10_-2, S-CAG_10_-3 and S-CAG_10_-4) fitted to two FRET states due to the branchpoint flexibility (as shown in C). The dotted lines between panels B and D connect the major FRET peak in the interrupted 3WJs with a FRET population in the static control structures. Panel D is adapted from (33) (https://creativecommons.org/licenses/by/4.0/).

We reported previously a mobile (CAG)_10_ 3WJ and four static (CAG)_10_ 3WJs, which were designed as controls for the 4 positional isomers that could form in the mobile 3WJ through branch migration (33,34). The FRET histogram for the pure (CAG)_10_ structure is shown in Figure 3A and displays FRET states across the whole FRET range, with six states required for adequate fitting, in addition to the zero-FRET peak, which is observed in all single-molecule FRET histograms due to acceptor bleaching or blinking (full MFD plot in Supplementary Figure S1). Although each interrupted 3WJ could, in principle, populate all of the possible positional isomers (Figure 2), we find that fewer FRET states are required to fit the data (Figure 3B), and that there is one major population in each case. As the position of the interrupt is moved, the major FRET population corresponds to the value expected for the positional isomer in which the interrupt is located in the hairpin loop (Figure 2). In CAA-1, for example, the positional isomer without a CA mismatch is on the left of Figure 2A and places the FRET dyes in close proximity. For this sample, the observed major FRET peak is at a high FRET efficiency, E = 0.80 (Figure 3B and Supplementary Table S1). For CAA-2, CAA-3 and CAA-4, the mismatch-free isomer (Figure 2B–D) would result in an increase in the dye-dye distance, agreeing with the observed major populations at *E* = 0.47, 0.08 and 0.04, respectively.

As shown previously, the four static (CAG)_10_ control 3WJs show two FRET states which we attributed to a local conformational flexibility at the branchpoint (Figure 3C) (33), based on earlier work with non-repeat 3WJs (36). Each of the major FRET populations in the interrupted structures is in good agreement with the FRET states observed in the corresponding static 3WJ (Figure 3D), showing that the positional isomer that positions the CAA in the loop is preferred (Figure 3B and Supplementary Table S1). Differences between the static and mobile 3WJs are attributed to the local sequence variation at the branchpoint. It is also worth noting that two small high FRET populations are found in CAA-2–4, suggesting that this positional isomer, with two populations due to branchpoint folding, (Figure 3C) is more stable than the other isomers with CA mismatches.

#### TIRF of surface-immobilized (CAG)_10_ 3WJs with interrupts

**Figure 4.**
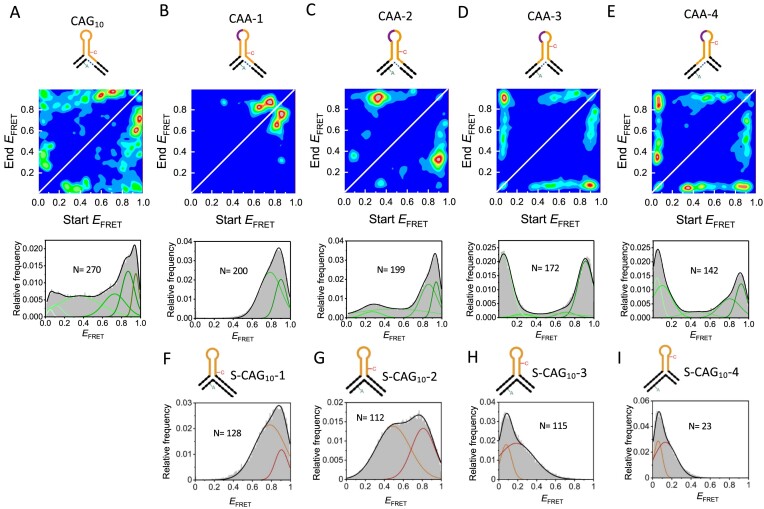
TIRF smFRET of (CAG)_10_ 3WJs containing CAA interrupts. (A–E) Transition density plots from unstitched TIRF time traces (top) and the corresponding *E*_FRET_ histograms (bottom) of a mobile (CAG)_10_ 3WJ with no interrupts (**A**) and with interrupts, CAA-1 (**B**), CAA-2 (**C**), CAA-3 (**D**) and CAA-4 (**E**).(F–I) *E*_FRET_ histograms for the static 3WJs S-CAG_10_-1 (**F**), S-CAG_10_-2 (**G**), S-CAG_10_-3 (**H**) and S-CAG_10_-4 (**I**). The number of single molecules (N) measured is indicated in each panel. Panels (F)–(I) are adapted from (33) (https://creativecommons.org/licenses/by/4.0/).

To investigate whether the FRET states observed in the MFD data (above) are interconverting, we conducted TIRF experiments with 3WJs that were immobilized to a PEG-coated coverslip via a biotin on the 5′ end of the long DNA strand. We observed clear anti-correlated changes in the donor and acceptor signals for all samples indicative of dynamic structural changes (Supplementary Figure S2). Transition density plots (TDPs), which show the numbers of transitions between each FRET state, illustrate these dynamics (Figure 4). As described previously, the 3WJ with a pure (CAG)_10_ slipout (Figure 4A) is interconverting between 6 resolvable FRET states (34). For the 3WJs with interrupts, the FRET efficiencies observed for TIRF were in good agreement with the MFD results (Supplementary Table S1), with a major FRET population that corresponds to the trapping of the interrupt in the hairpin loop. As observed in the confocal data, there is also a high-FRET population in all samples. The TDPs for the interrupted 3WJs (Figure 4B–E) clearly show that only a small number of FRET states are occupied, with most transitions occurring between the major conformation and the high FRET state. The rates of transitions are discussed in a subsequent section.

### Single-molecule spectroscopy of (CAG)_11_ 3WJs with and without a CAA interrupt

To assess the influence of repeat parity on interrupted structure dynamics, we conducted experiments with a pure mobile (CAG)_11_ 3WJ, and a mobile (CAG)_11_ 3WJ with a CAA interrupt. We also designed four static (CAG)_11_ 3WJs without interrupts (Supplementary Figure S3) as controls to mimic the positional isomers that could occur via branch migration in the mobile (CAG)_11_ 3WJ. Apart from the addition of a CAG repeat, the only other difference to the (CAG)_10_ repeats is that the acceptor dye is positioned further from the branchpoint.

The confocal (Supplementary Figure S4) and TIRF (Supplementary Figure S5) smFRET measurements for the static samples show the expected overall shift in FRET as the position of the slipout moves along the duplex. One difference from the static (CAG)_10_ samples (33) is that there are more FRET states observed (Supplementary Table S2). Whereas the even (CAG)_10_ static samples showed two-state behaviour, up to four states are needed to fit the odd (CAG)_11_ 3WJs. We attribute this to a competition between alternative slipout structures, possibly due to an equilibrium between triloop and tetraloop conformations (29). Nonetheless, the general shift in FRET demonstrates that migration between positional isomers in the mobile 3WJs should be discernible.

**Figure 5.**
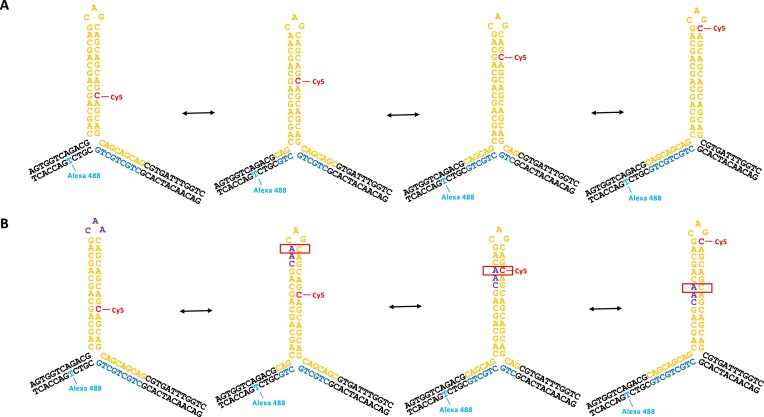
Possible stable positional isomers following branch migration for a (CAG)_11_ hairpin with and without a CAA interrupt. Illustrations showing possible stable conformations due to branchpoint migration for 3WJs with eleven CAG repeats in the hairpin for either a pure repeat (**A**) or with a CAA interrupt in the sixth triplet from the 5′ end (**B)**. CA mismatches are highlighted (red box).

**Figure 6.**
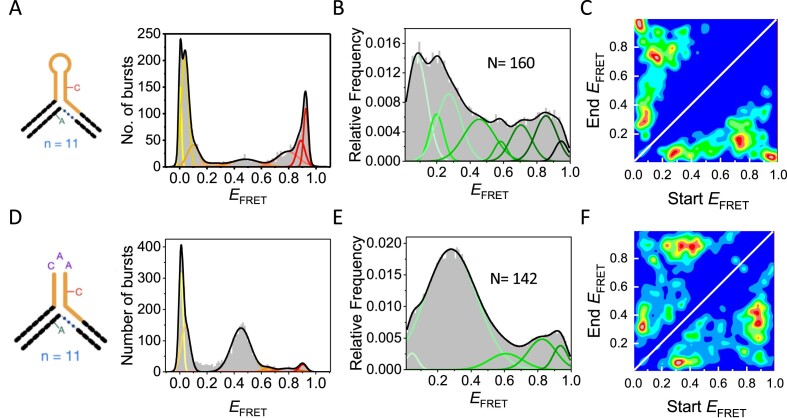
Confocal and TIRF smFRET of (CAG)_11_ 3WJs with and without a CAA interrupt. (**A**) Confocal *E*_FRET_ histograms of mobile (CAG)_11_ 3WJs. (**B**) *E*_FRET_ histograms from TIRF microscopy of mobile (CAG)_11_ 3WJs. (**C**) Corresponding transitions density plots. The best model was found to be (*J*_opt_) = 8, with *E*_1_ = 0.07, *E*_2_ = 0.17, *E*_3_ = 0.29, *E*_4_ = 0.41, *E*_5_ = 0.55, *E*_6_ = 0.79, *E*_7_ = 0.89 and *E*_8_ = 0.93. (**D**–**F**) Equivalent data as in A–C using (CAG)_5_(CAA)(CAG)_5_, where the interrupt is positioned in the loop. The data best fit a 5-state model, with *E*_1_ = 0.11, *E*_2_ = 0.29, *E*_3_ = 0.44, *E*_4_ = 0.69 and *E*_5_ = 0.88. The number of single molecules (N) measured is indicated in each panel.

For the pure (CAG)_11_ 3WJ (Figure 5A), the confocal and TIRF smFRET results are broadly similar to the pure (CAG)_10_ 3WJ (Supplementary Figures S6 and S7). The pure (CAG)_11_ repeat explores up to eight resolvable FRET states as observed with MFD and TIRF (Figure 6A, B and Supplementary Table S3), with interconversion between all of these states (Figure 6C), which we attribute to free branch migration between positional isomers and alternative folding conformations for each isomer (33). The greater number of FRET states for the odd versus even 3WJ with pure slipout mirrors the greater complexity observed in the static samples (see above). For the (CAG)_11_ with an interrupt (Figure 5B), there are fewer states than with the pure 3WJ, indicative of reduced configurational freedom (Supplementary Figures S6 and S7). The major FRET state has *E* = ∼0.45 (Figure 6D, E and Supplementary Table S4) and, by comparing with the static control 3WJs (Supplementary Figures S3A, B and S4A, B), the five FRET states observed are consistent with conformations that place the interrupt in or next to the hairpin loop (Figure 5A–B). Although there are fewer FRET states for (CAG)_11_ 3WJ with the interrupt, the greater configurational freedom in comparison to the (CAG)_10_ 3WJs with interrupts is clear from the TDP (Figure 6F); the TDP for the interrupted (CAG)_11_ 3WJ (Figure 6F) is qualitatively more like that of the odd or even 3WJ without an interrupt (Figures 4A and 6C, respectively). The other major difference between the samples with and without interrupts is in the magnitude of the interconversion rates, which we present in the next section.

### Kinetics of branch migration for 3WJs containing a CAA interrupt

**Figure 7.**
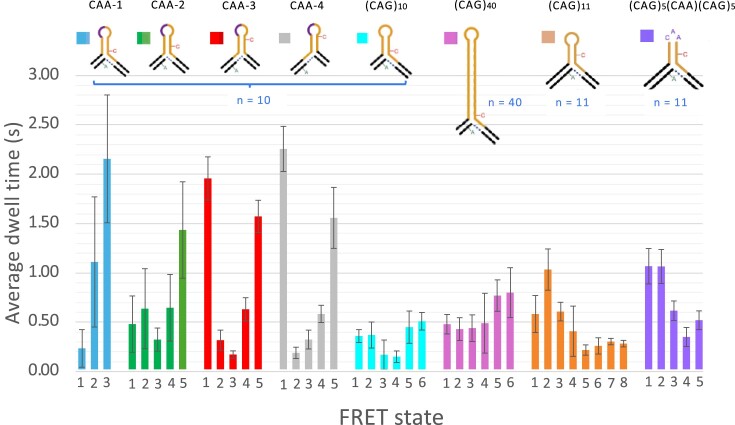
Dwell times for each FRET state in the 3WJs. From the TIRF data, cumulative dwell-time distributions for each FRET state were fit to biexponential decays (Supplementary Figures S8–S10) from which an average dwell time was computed. See Supplementary Tables S1-S4 for the FRET efficiency of each state. Data for mobile (CAG)_10_ and (CAG)_40_ were reported previously (34). The errors bars were determined from an estimate of the variability of biexponential fitting parameters across the sample by BOBA-FRET analysis (see Materials and methods).

From the TIRF data, we are able to extract the rates for transitions between each FRET state using methods described previously (Supplementary Figures S8-S10, Supplementary Tables S5 and S6) (34). The cumulative dwell-time distributions were fit to biexponential decays. We calculated average dwell times from these decays (Figure 7), which correspond to the average time spent in a particular FRET state before transiting to any of the other states. We have analysed the average dwell times for the new mobile 3WJ samples and compared them with those already reported for the mobile (CAG)_10_ 3WJ and an equivalent 3WJ with a (CAG)_40_ slipout (Figure 7).

The most noticeable feature of the dwell-time data is that the presence of a CAA interrupt in the (CAG)_10_ 3WJ results in one or two transitions that are much slower than any of the transitions observed for the pure (CAG)_10_ repeat, with dwell times ∼4 times longer than for any transition found for the pure (CAG)_10_ repeat and ∼ 3 times as long as for the (CAG)_40_ repeat. For CAA-1, the two long-lived states correspond to the isomer that has the interrupt in the hairpin loop. For CAA-3 and CAA-4, one of the long-lived states corresponds to the low-FRET isomer that has the interrupt in the loop, while the other one is for a high-FRET state. For CAA-2, the longest lived state is for the high-FRET state, which corresponds to the positional isomer with a CA mismatch adjacent to the hairpin loop.

Therefore, adding a single, mismatched nucleotide has suppressed the dynamics for certain conformations and these are predominantly associated with trapping of the interrupt in or adjacent to the loop. The prevalence of the high-FRET state for CAA-3 and CAA-4 may be due to some additional stabilizing effect resulting from the non-repeat sequence adjacent to the repeat. Another possibility is that the proximity of the dyes to the branchpoint in the high-FRET state may be stabilising this conformation. However, this seems unlikely because such a preference for the high-FRET state is not seen in the pure (CAG)_10_ 3WJ, where the dyes are just as close to the branchpoint. In contrast to the even 3WJs, adding the interrupt to the odd-parity (CAG)_11_ 3WJ has little effect on the dwell times, relative to the pure (CAG)_11_ 3WJ. However, the dwell times for the odd 3WJs are slightly longer on average than the pure (CAG)_10_ 3WJ (0.46 s versus 0.34 s). As discussed above, the FRET data for the (CAG)_11_ 3WJ with an interrupt are consistent with trapping of the 3WJ in two of the positional isomers (with interrupt both in and near the branchpoint), so we attribute the faster dynamics for the odd interrupted 3WJ to local interconversion between these two trapped states.

## Discussion

While the mechanism of repeat expansion in REDs is still unknown, most models assume that it involves non-canonical secondary DNA structure intermediates that are associated with the repeat sequence. Recent advances have confirmed the presence of such secondary structures *in vivo* and their correlation with genetic instability (48). Single-molecule methods are ideally suited for studying systems with static or dynamic heterogeneity, and a number of studies have recently applied single-molecule fluorescence techniques to study DNA repeats, in free hairpins and in the context of a 3WJ. In both cases, it is the self-complementarity of the repeat sequence that allows a stable hairpin to form (15).

Slippage by up to two repeat units has been observed previously for a number of repeat hairpin structures, including CAG repeats (26,27). Important roles were found for the loop structure, with CAG favouring a AGCA tetraloop. Placing an interrupt in the hairpin resulted in suppression of dynamics due to the preferential location of the interrupt in the hairpin loop, in agreement with earlier ensemble studies (28).

Here, we have studied an even and odd CAG repeat sequence within a 3WJ, with and without a single CAA interrupt. We showed previously that a stable CAG or CTG hairpin slipout was able to migrate along a duplex, complementing earlier ensemble work with small repeat loops (40–42). We observed migration by up to four repeats, which was the maximum allowed by our constructs. We find here that the presence of an interrupt dramatically reduces the number of accessible conformations. For the even repeat 3WJs, the interrupt is preferentially located in or near the hairpin loop with dynamics that are markedly slower than for the pure repeat, and slower even than for a pure repeat 3WJ with an additional 30 CAG repeats. In contrast, the odd 3WJs show greater conformational flexibility, which we attribute to trapping in two states that can interconvert, possibly driven by competition between triloop and tetraloop conformations (29).

Although slipping has now been observed at the single-molecule level for both hairpins and 3WJs, and both structures may be important for expansion, it is important to note some of the differences between these structures. A free hairpin produced by nicking of a 3WJ has a global conformational flexibility that is more akin to a replication fork (49). In contrast, fixing the hairpin at both ends within the context of an intact 3WJ leads to a constrained branchpoint that can result in the unpairing of bases or branchpoint re-arrangement (35,36,50). In hairpins, CAG tetraloops were found to be favoured for both odd and even hairpins, with triloops disfavoured (29). However, for an odd hairpin to accommodate a tetraloop, a CAG overhang is required. In an intact 3WJ, where the repeat is placed within the context of a non-repeat DNA duplex, the production of such overhangs is not possible without either introducing a bulge elsewhere in the structure, or by nicking one strand.

Error-prone repair has emerged as a leading contender for the cause of expansion in REDs, whereby repeat-associated secondary structures are thought to result in mis-direction of repair and/or error-prone re-synthesis (51). Depending on the repair mechanism, migration could allow loops on opposing strands (e.g. CAG and CTG) to move far enough apart for processing to result in expansion, as proposed earlier (52). Alternatively, it may be that it is the migrating slipout itself that is the target, as suggested by the effect of abasic lesions on base excision repair (42). Such processes may be impeded by the presence of an interrupt (34). In a model presented recently (53), a reduced configurational space would prevent expansion by limiting the extent of branch migration. Our data support this model, whereby an interrupt would split the repeat into two pure repeat tracts, with the longer of those becoming the effective expansion-prone repeat length (53).

## Supplementary Material

gkae644_Supplemental_File

## Data Availability

The data underlying this article are available from the University of Glasgow data repository Enlighten at https://doi.org/10.5525/gla.researchdata.1609.
