# Supplementary material for: A single CAA interrupt in a DNA three-way junction containing a CAG repeat hairpin results in parity-dependent trapping
Source: Nucleic Acids Res. 2024 Jul 23;52(15):9317–27. doi: 10.1093/nar/gkae644 (PMC11347167; doi:10.1093/nar/gkae644)
Supplement: gkae644_Supplemental_File [file gkae644_supplemental_file.pdf]

Supplementary Data

A single CAA interrupt in a DNA three-way junction containing a CAG repeat hairpin results in parity-dependent trapping

Gillian M. Cadden, Svea J. Wilken and Steven W. Magennis*

School of Chemistry, University of Glasgow, Joseph Black Building, University Avenue, Glasgow, G12 8QQ, UK

* To whom correspondence should be addressed. Email: steven.magennis@glasgow.ac.uk

This file contains:

Oligonucleotide sequences

Supplementary Tables 1-6

Supplementary Figures 1-10

Oligonucleotide sequences

The top and bottom strands are named according to the orientation of the 3WJ structures, as drawn in the main text and supplementary data.

The static and mobile $(CAG)_{10}$ oligonucleotides below with pure repeats were described previously (1,2).

a) **Static $(CAG)_{10}$ 3WJs**

X = C-Cy5 and Y = T-Alexa488

S-CAG$_{10}$-1

Top: 5’ AGT GGT CAG ACG CAG CAG CAG CAG CAG CAG CAG CAG CAG **X**AG CTC GGC ACT CGT GAT TTG GTC

Bottom: 5' /Biotin/ GAC CAA ATC ACG AGT GCC GAG CGT C**Y**G ACC ACT

S-CAG$_{10}$-2

Top: 5' AGT GGT CAG ACG ATG CAG CAG CAG CAG CAG CAG CAG CAG **X**AG CAG CTC ACT CGT GAT TTG GTC

Bottom: 5' / Biotin/ GAC CAA ATC ACG AGT GAG CAT CGT C**Y**G ACC ACT

S-CAG$_{10}$-3

Top: 5' AGT GGT CAG ACG ACT ATG CAG CAG CAG CAG CAG CAG CAG **X**AG CAG CAG CTC CGT GAT TTG GTC

Bottom: 5' /Biotin/ GAC CAA ATC ACG GAG CAT AGT CGT C**Y**G ACC ACT

S-CAG$_{10}$-4

Top: 5' AGT GGT CAG ACG ACT GGC ATG CAG CAG CAG CAG CAG CAG **X**AG CAG CAG CAG CGT GAT TTG GTC

Bottom: 5' /Biotin/ GAC CAA ATC ACG CAT GCC AGT CGT C**Y**G ACC ACT

b) **Static $(CAG)_{11}$ 3WJs**

X = C-Cy5 and Y = T-Alexa488

S-CAG$_{11}$-1

Top: 5’ AGT GGT CAG ACG CAG CAG CAG CAG CAG CAG CAG CAG CAG XAG CAG CTC GGC ACT CGT GAT TTG GTC

Bottom: 5' /Biotin/ GAC CAA ATC ACG AGT GCC GAG CGT C**Y**G ACC ACT

S-CAG$_{11}$-2

Top: 5’ AGT GGT CAG ACG ATG CAG CAG CAG CAG CAG CAG CAG CAG XAG CAG CAG CTC ACT CGT GAT TTG GTC

Bottom: 5' / Biotin/ GAC CAA ATC ACG AGT GAG CAT CGT C**Y**G ACC ACT

S-CAG$_{11}$-3

Top: 5’ AGT GGT CAG ACG ACT ATG CAG CAG CAG CAG CAG CAG CAG XAG CAG CAG CAG CTC CGT GAT TTG GTC

Bottom: 5' /Biotin/ GAC CAA ATC ACG GAG CAT AGT CGT C**Y**G ACC ACT

S-CAG$_{11}$-4

Top: 5’ AGT GGT CAG ACG ACT GGC ATG CAG CAG CAG CAG CAG CAG XAG CAG CAG CAG CAG CGT GAT TTG GTC

Bottom: 5' /Biotin/ GAC CAA ATC ACG CAT GCC AGT CGT C**Y**G ACC ACT

c) **Mobile 3WJs**

X = C-Cy5 and Y = T-Alexa488

(CAG)$_{10}$

Top: 5' AGT GGT CAG ACG CAG CAG CAG CAG CAG CAG CAG CAG CAG **X**AG CAG CAG CAG CGT GAT TTG GTC

Bottom: 5’ /Biotin/ GAC CAA ATC ACG CTG CTG CTG CGT C**Y**G ACC ACT

(CAG)$_{40}$

Top: 5' AGT GGT CAG ACG CAG CAG CAG CAG CAG CAG CAG CAG CAG CAG CAG CAG CAG CAG CAG CAG CAG CAG CAG CAG CAG CAG CAG CAG CAG CAG CAG CAG CAG CAG CAG CAG CAG CAG CAG CAG CAG CAG CAG CAG **X**AG CAG CAG CAG CGT GAT TTG GTC

Bottom: 5’ /Biotin/ GAC CAA ATC ACG CTG CTG CTG CGT C**Y**G ACC ACT

(CAG)$_{11}$

Top: 5' AGT GGT CAG ACG CAG CAG CAG CAG CAG CAG CAG CAG CAG XAG CAG CAG CAG CAG CGT GAT TTG GTC

Bottom: 5’ /Biotin/ GAC CAA ATC ACG CTG CTG CTG CGT C**Y**G ACC ACT

d) **Mobile 3WJs with CAA interrupts**

X = C-Cy5 and Y = T-Alexa488

CAA-1

Top: 5' AGT GGT CAG ACG CAG CAG CAG CAG CAA CAG CAG CAG CAG XAG CAG CAG CAG CGT GAT TTG GTC

Bottom: 5' /Biotin/ GAC CAA ATC ACG CTG CTG CTG CGT C**Y**G ACC ACT

CAA-2

Top: 5' AGT GGT CAG ACG CAG CAG CAG CAG CAG CAA CAG CAG CAG XAG CAG CAG CAG CGT GAT TTG GTC

Bottom: 5' /Biotin/ GAC CAA ATC ACG CTG CTG CTG CGT C**Y**G ACC ACT

CAA-3

Top: 5' AGT GGT CAG ACG CAG CAG CAG CAG CAG CAG CAA CAG CAG XAG CAG CAG CAG CGT GAT TTG GTC

Bottom: 5' /Biotin/ GAC CAA ATC ACG CTG CTG CTG CGT C**Y**G ACC ACT

CAA-4

Top: 5' AGT GGT CAG ACG CAG CAG CAG CAG CAG CAG CAG CAA CAG XAG CAG CAG CAG CGT GAT TTG GTC

Bottom: 5' /Biotin/ GAC CAA ATC ACG CTG CTG CTG CGT C**Y**G ACC ACT

$(CAG)_5(CAA)(CAG)_5$

Top: 5' AGT GGT CAG ACG CAG CAG CAG CAG CAG CAA CAG CAG CAG XAG CAG CAG CAG CAG CGT GAT TTG GTC

Bottom: 5' /Biotin/ GAC CAA ATC ACG CTG CTG CTG CGT C**Y**G ACC ACT

Supplementary Tables

Supplementary Table 1: FRET levels for mobile $(CAG)_{10}$ 3WJs containing a CAA interrupt calculated by MFD and TIRF.

FRET state	1				2				3				4				5			
method	MFD			TIRF	MFD			TIRF	MFD			TIRF	MFD			TIRF	MFD			TIRF
CAA-1	0.46 (0.15)			0.35 (0.02)	0.80 (0.14)			0.69 (0.02)	0.89 (0.07)			0.84 (0.01)	n/a			n/a	n/a			n/a
	w	A	H		w	A	H		w	A	H									
	0.3	5.3	14.6		0.2	30.5	157.7		0.1	20.2	132.4									
CAA-2	0.27 (0.25)			0.12 (0.04)	0.47 (0.01)			0.31 (0.05)	0.72 (0.10)			0.53 (1.06)	0.87 (0.06)			0.77 (0.07)	0.92 (0.01)			0.90 (0.05)
	w	A	H		w	A	H		w	A	H		w	A	H		w	A	H	
	0.4	15.3	32.6		0.1	34.1	245.3		0.2	12.4	42.4		0.06	4.49	59.7		0.04	6.1	130.4	
CAA-3	0.08 (0.01)			0.09 (0.07)	n/a			0.27 (0.04)	0.39 (0.29)			0.49 (0.05)	0.84 (0.04)			0.73 (0.05)	0.91 (0.01)			0.89 (0.06)
	w	A	H						w	A	H		w	A	H		w	A	H	
	0.1	45.7	498.0						1.0	9.0	7.6		0.11	6.16	45.9		0.1	3.7	62.5	
CAA-4	0.04 (0.01)			0.08 (0.05)	n/a			0.34 (0.05)	n/a			0.52 (0.04)	0.79 (0.05)			0.75 (0.05)	0.90 (0.01)			0.89 (0.04)
	w	A	H										w	A	H		w	A	H	
	0.01	58.4	818.9										0.2	6.8	26.3		0.1	4.3	54.9	

E values from MFD were obtained from Gaussian fits of the FRET histogram with width (w), area (A) and height (H). For TIRF, E values were determined through clustering analysis of the transition density plots in MASH-FRET (3).

Supplementary table 2: FRET levels for static $(CAG)_{11}$ 3WJs calculated by MFD and TIRF.

<table>
<tr><td>FRET state</td><td colspan="4">1</td><td colspan="4">2</td><td colspan="4">3</td><td colspan="4">4</td></tr>
<tr><td>method</td><td colspan="3">MFD</td><td>TIRF</td><td colspan="3">MFD</td><td>TIRF</td><td colspan="3">MFD</td><td>TIRF</td><td colspan="3">MFD</td><td>TIRF</td></tr>
<tr><td rowspan="3">S_{11}-1</td><td colspan="3">0.18 (0.14)</td><td rowspan="3">0.18 (0.05)</td><td colspan="3">0.48 (0.04)</td><td rowspan="3">0.35 (0.05)</td><td colspan="3">0.79 (0.02)</td><td rowspan="3">0.63 (0.06)</td><td colspan="3">0.89 (0.01)</td><td rowspan="3">0.87 (0.04)</td></tr>
<tr><td>w</td><td>A</td><td>H</td><td>w</td><td>A</td><td>H</td><td>w</td><td>A</td><td>H</td><td>w</td><td>A</td><td>H</td></tr>
<tr><td>0.4</td><td>29.7</td><td>108.5</td><td>0.2</td><td>15.8</td><td>88.4</td><td>0.2</td><td>50.4</td><td>185.8</td><td>0.1</td><td>24.1</td><td>299.4</td></tr>
<tr><td rowspan="3">S_{11}-2</td><td colspan="3">0.08 (0.01)</td><td rowspan="3">0.13 (0.03)</td><td colspan="3">0.39 (0.01)</td><td rowspan="3">0.36 (0.04)</td><td colspan="3">0.58 (0.01)</td><td rowspan="3">0.57 (0.03)</td><td colspan="3">0.71 (0.03)</td><td rowspan="3">0.89 (0.04)</td></tr>
<tr><td>w</td><td>A</td><td>H</td><td>w</td><td>A</td><td>H</td><td>w</td><td>A</td><td>H</td><td>w</td><td>A</td><td>H</td></tr>
<tr><td>0.1</td><td>17.7</td><td>131.2</td><td>0.3</td><td>18.5</td><td>56.0</td><td>0.04</td><td>0.4</td><td>9.9</td><td>0.2</td><td>5.2</td><td>23.0</td></tr>
<tr><td rowspan="3">S_{11}-3</td><td colspan="3">0.06 (0.01)</td><td rowspan="3">0.08 (0.03)</td><td colspan="3">0.14 (0.03)</td><td rowspan="3">0.30 (0.04)</td><td colspan="3" rowspan="3">n/a</td><td rowspan="3">0.84 (0.04)</td><td colspan="3" rowspan="3">n/a</td><td rowspan="3">n/a</td></tr>
<tr><td>w</td><td>A</td><td>H</td><td>w</td><td>A</td><td>H</td></tr>
<tr><td>0.1</td><td>62.8</td><td>633.8</td><td>0.2</td><td>31.3</td><td>170.1</td></tr>
<tr><td>S_{11}-4</td><td colspan="3">n/a</td><td>0.15 (0.02)</td><td colspan="3">n/a</td><td>n/a</td><td colspan="3">n/a</td><td>n/a</td><td colspan="3">n/a</td><td>n/a</td></tr>
</table>

E values from MFD were obtained from Gaussian fits of the FRET histogram with width (w), area (A) and height (H). For TIRF, E values were determined through clustering analysis of the transition density plots in MASH-FRET (3).

Supplementary table 3: FRET levels for mobile pure $(CAG)_{11}$ calculated by MFD and TIRF.

<table>
<tr><td>FRET state</td><td colspan="4">1</td><td colspan="4">2</td><td colspan="4">3</td><td colspan="4">4</td><td colspan="2">5</td></tr>
<tr><td>Method</td><td colspan="3">MFD</td><td>TIRF</td><td colspan="3">MFD</td><td>TIRF</td><td colspan="3">MFD</td><td>TIRF</td><td colspan="3">MFD</td><td>TIRF</td><td>MFD</td><td>TIRF</td></tr>
<tr><td rowspan="3">(CAG)11</td><td colspan="3">0.04 (0.01)</td><td rowspan="3">0.07 (0.05)</td><td colspan="3">0.10 (0.03)</td><td rowspan="3">0.17 (0.05)</td><td colspan="3">0.24 (0.12)</td><td rowspan="3">0.29 (0.05)</td><td colspan="3">0.48 (0.03)</td><td rowspan="3">0.41 (0.05)</td><td rowspan="3">n/a</td><td rowspan="3">0.55 (0.05)</td></tr>
<tr><td>w</td><td>A</td><td>H</td><td>w</td><td>A</td><td>H</td><td>w</td><td>A</td><td>H</td><td>w</td><td>A</td><td>H</td></tr>
<tr><td>0.1</td><td>13.1</td><td>204.3</td><td>0.1</td><td>4.4</td><td>41.0</td><td>0.15</td><td>1.2</td><td>6.4</td><td>0.19</td><td>3.4</td><td>14.1</td></tr>
</table>

<table>
<tr><td>FRET state</td><td colspan="4">6</td><td colspan="4">7</td><td colspan="4">8</td></tr>
<tr><td>Method</td><td colspan="3">MFD</td><td>TIRF</td><td colspan="3">MFD</td><td>TIRF</td><td colspan="3">MFD</td><td>TIRF</td></tr>
<tr><td rowspan="3">(CAG)11</td><td colspan="3">0.79 (0.03)</td><td rowspan="3">0.70 (0.05)</td><td colspan="3">0.89 (0.02)</td><td rowspan="3">0.80 (0.05)</td><td colspan="3">0.93 (0.01)</td><td rowspan="3">0.93 (0.05)</td></tr>
<tr><td>w</td><td>A</td><td>H</td><td>w</td><td>A</td><td>H</td><td>w</td><td>A</td><td>H</td></tr>
<tr><td>0.2</td><td>5.6</td><td>28.6</td><td>0.1</td><td>3.7</td><td>49.4</td><td>0.03</td><td>4.6</td><td>109.2</td></tr>
</table>

E values from MFD were obtained from Gaussian fits of the FRET histogram with width (w), area (A) and height (H). For TIRF, E values were determined through clustering analysis of the transition density plots in MASH-FRET (3).

Supplementary table 4: FRET levels for mobile $(CAG)_{11}$ with a CAA interrupt calculated by MFD and TIRF.

FRET state	1				2				3				4				5			
Method	MFD			TIRF	MFD			TIRF	MFD			TIRF	MFD			TIRF	MFD			TIRF
$(CAG)_5(CAA)(CAG)_5$	0.10 (0.07)			0.11 (0.04)	0.32 (0.05)			0.29 (0.04)	0.46 (0.01)			0.44 (0.04)	0.76 (0.10)			0.69 (0.05)	0.90 (0.02)			0.88 (0.04)
	w	A	H		w	A	H		w	A	H		w	A	H		w	A	H	
	0.2	6.1	29.7		0.1	5.5	32.3		0.1	17.7	152.1		0.1	1.7	9.5		0.1	2.1	26.6	

E values from MFD were obtained from Gaussian fits of the FRET histogram with width (w), area (A) and height (H). For TIRF, E values were determined through clustering analysis of the transition density plots in MASH-FRET (3).

Supplementary table 5: State-to-state kinetics for mobile $(CAG)_{10}$ 3WJs with CAA interrupt.

CAA-1

Transition	Relative population	τj (s)	τjj (s)	τ (bi)1 (s)	τ (bi)2 (s)	A1 (%)	A2 (%)	τ (bi)av (s)
1 to 2	0.02	0.78 (0.28)	0.26 (0.09)	0.07 (0.01)	0.66 (0.16)	72.09 (8.23)	27.91 (8.23)	0.23 (0.19)
1 to 3	0.04	0.78 (0.28)	0.52 (0.19)	0.07 (0.01)	0.66 (0.16)	72.09 (8.23)	27.91 (8.23)	0.23 (0.19)
2 to 1	0.02	1.65 (0.13)	0.07 (0.01)	0.40 (0.09)	3.10 (0.37)	73.62 (4.15)	26.38 (4.15)	1.11 (0.66)
2 to 3	0.44	1.65 (0.13)	1.58 (0.12)	0.40 (0.09)	3.10 (0.37)	73.62 (4.15)	26.38 (4.15)	1.11 (0.66)
3 to 1	0.04	2.15 (0.27)	0.14 (0.02)	0.97 (0.08)	3.76 (0.25)	57.53 (7.09)	42.47 (7.09)	2.16 (0.65)
3 to 2	0.44	2.15 (0.27)	1.51 (0.19)	0.97 (0.08)	3.76 (0.25)	57.53 (7.09)	42.47 (7.09)	2.16 (0.65)

CAA-2

Transition	Relative population	τj (s)	τjj (s)	τ (bi)1 (s)	τ (bi)2 (s)	A1 (%)	A2 (%)	τ (bi)av (s)
1 to 2	0.06	0.94 (0.20)	0.35 (0.07)	0.15 (0.04)	2.23 (0.85)	83.93 (11.15)	16.07 (11.15)	0.48 (0.29)
1 to 3	0.03	0.94 (0.20)	0.20 (0.04)	0.15 (0.04)	2.23 (0.85)	83.93 (11.15)	16.07 (11.15)	0.48 (0.29)
1 to 5	0.02	0.94 (0.20)	0.12 (0.02)	0.15 (0.04)	2.23 (0.85)	83.93 (11.15)	16.07 (11.15)	0.48 (0.29)
1 to 4	0.04	0.94 (0.20)	0.28 (0.06)	0.15 (0.04)	2.23 (0.85)	83.93 (11.15)	16.07 (11.15)	0.48 (0.29)
2 to 1	0.05	0.37 (0.02)	0.07 (0.004)	0.22 (0.06)	2.22 (1.41)	79.08 (16.24)	20.92 (16.24)	0.64 (0.41)
2 to 3	0.02	0.37 (0.02)	0.03 (0.002)	0.22 (0.06)	2.22 (1.41)	79.08 (16.24)	20.92 (16.24)	0.64 (0.41)
2 to 4	0.04	0.37 (0.02)	0.06 (0.003)	0.22 (0.06)	2.22 (1.41)	79.08 (16.24)	20.92 (16.24)	0.64 (0.41)
2 to 5	0.16	0.37 (0.02)	0.22 (0.01)	0.22 (0.06)	2.22 (1.41)	79.08 (16.24)	20.92 (16.24)	0.64 (0.41)
3 to 1	0.03	1.00 (0.10)	0.27 (0.03)	0.08 (0.01)	2.19 (0.37)	88.39 (2.41)	11.61 (2.41)	0.32 (0.12)
3 to 2	0.03	1.00 (0.10)	0.21 (0.02)	0.08 (0.01)	2.19 (0.37)	88.39 (2.41)	11.61 (2.41)	0.32 (0.12)
3 to 4	0.01	1.00 (0.10)	0.07 (0.01)	0.08 (0.01)	2.19 (0.37)	88.39 (2.41)	11.61 (2.41)	0.32 (0.12)
3 to 5	0.06	1.00 (0.10)	0.45 (0.05)	0.08 (0.01)	2.19 (0.37)	88.39 (2.41)	11.61 (2.41)	0.32 (0.12)
4 to 1	0.02	1.11 (0.07)	0.17 (0.01)	0.08 (0.03)	1.55 (0.18)	61.72 (5.45)	38.28 (5.45)	0.65 (0.34)
4 to 2	0.04	1.11 (0.07)	0.40 (0.03)	0.08 (0.03)	1.55 (0.18)	61.72 (5.45)	38.28 (5.45)	0.65 (0.34)
4 to 3	0.01	1.11 (0.07)	0.09 (0.01)	0.08 (0.03)	1.55 (0.18)	61.72 (5.45)	38.28 (5.45)	0.65 (0.34)
4 to 5	0.05	1.11 (0.07)	0.46 (0.03)	0.08 (0.03)	1.55 (0.18)	61.72 (5.45)	38.28 (5.45)	0.65 (0.34)
5 to 1	0.02	1.58 (0.12)	0.10 (0.01)	0.63 (0.12)	2.19 (0.25)	48.36 (9.36)	51.64 (9.36)	1.44 (0.49)
5 to 2	0.15	1.58 (0.12)	0.83(0.06)	0.63 (0.12)	2.19 (0.25)	48.36 (9.36)	51.64 (9.36)	1.44 (0.49)
5 to3	0.06	1.58 (0.12)	0.35 (0.03)	0.63 (0.12)	2.19 (0.25)	48.36 (9.36)	51.64 (9.36)	1.44 (0.49)
5 to 4	0.05	1.58 (0.12)	0.29 (0.02)	0.63 (0.12)	2.19 (0.25)	48.36 (9.36)	51.64 (9.36)	1.44 (0.49)

CAA-3

Transition	Relative population	τj (s)	τjj (s)	τ (bi)1 (s)	τ (bi)2 (s)	A1 (%)	A2 (%)	τ (bi)av (s)
1 to 2	0.04	2.38 (0.13)	0.31 (0.02)	0.66 (0.11)	3.23 (0.24)	49.65 (5.22)	50.35 (5.22)	1.96 (0.22)
1 to 3	0.06	2.38 (0.13)	0.49 (0.03)	0.66 (0.11)	3.23 (0.24)	49.65 (5.22)	50.35 (5.22)	1.96 (0.22)
1 to 4	0.07	2.38 (0.13)	0.58 (0.03)	0.66 (0.11)	3.23 (0.24)	49.65 (5.22)	50.35 (5.22)	1.96 (0.22)
1 to 5	0.12	2.38 (0.13)	1.00 (0.05)	0.66 (0.11)	3.23 (0.24)	49.65 (5.22)	50.35 (5.22)	1.96 (0.22)
2 to 1	0.04	0.62 (0.12)	0.30 (0.06)	0.05 (0.04)	1.16 (0.27)	76.23 (6.40)	23.77 (6.40)	0.32 (0.10)
2 to 3	0.01	0.62 (0.12)	0.05 (0.01)	0.05 (0.04)	1.16 (0.27)	76.23 (6.40)	23.77 (6.40)	0.32 (0.10)
2 to 4	0.01	0.62 (0.12)	0.10 (0.02)	0.05 (0.04)	1.16 (0.27)	76.23 (6.40)	23.77 (6.40)	0.32 (0.10)
2 to 5	0.02	0.62 (0.12)	0.18 (0.03)	0.05 (0.04)	1.16 (0.27)	76.23 (6.40)	23.77 (6.40)	0.32 (0.10)
3 to 1	0.06	0.57 (0.07)	0.27 (0.03)	0.03 (0.002)	1.29 (0.17)	88.70 (2.46)	11.30 (2.46)	0.17 (0.04)
3 to 2	0.01	0.57 (0.07)	0.03 (0.003)	0.03 (0.002)	1.29 (0.17)	88.70 (2.46)	11.30 (2.46)	0.17 (0.04)
3 to 4	0.01	0.57 (0.07)	0.06 (0.01)	0.03 (0.002)	1.29 (0.17)	88.70 (2.46)	11.30 (2.46)	0.17 (0.04)
3 to 5	0.05	0.57 (0.07)	0.22 (0.03)	0.03 (0.002)	1.29 (0.17)	88.70 (2.46)	11.30 (2.46)	0.17 (0.04)
4 to 1	0.07	1.25 (0.10)	0.40 (0.03)	0.10 (0.02)	2.04 (0.28)	72.56 (4.32)	27.44 (4.32)	0.63 (0.12)
4 to 2	0.01	1.25 (0.10)	0.07 (0.01)	0.10 (0.02)	2.04 (0.28)	72.56 (4.32)	27.44 (4.32)	0.63 (0.12)
4 to 3	0.02	1.25 (0.10)	0.10 (0.01)	0.10 (0.02)	2.04 (0.28)	72.56 (4.32)	27.44 (4.32)	0.63 (0.12)
4 to 5	0.11	1.25 (0.10)	0.68 (0.05)	0.10 (0.02)	2.04 (0.28)	72.56 (4.32)	27.44 (4.32)	0.63 (0.12)
5 to 1	0.11	1.80 (0.08)	0.64 (0.03)	0.25 (0.07)	2.04 (0.15)	26.10 (5.93)	73.90 (5.93)	1.57 (0.16)
5 to 2	0.02	1.80 (0.08)	0.13 (0.01)	0.25 (0.07)	2.04 (0.15)	26.10 (5.93)	73.90 (5.93)	1.57 (0.16)
5 to 3	0.05	1.80 (0.08)	0.33 (0.01)	0.25 (0.07)	2.04 (0.15)	26.10 (5.93)	73.90 (5.93)	1.57 (0.16)
5 to 4	0.12	1.80 (0.08)	0.70 (0.03)	0.25 (0.07)	2.04 (0.15)	26.10 (5.93)	73.90 (5.93)	1.57 (0.16)

CAA-4

Transition	Relative population	τj (s)	τjj (s)	τ (bi)1 (s)	τ (bi)2 (s)	A1 (%)	A2 (%)	τ (bi)av (s)
1 to 2	0.10	2.73 (0.16)	0.96 (0.06)	0.32 (0.07)	3.43 (0.22)	37.82 (5.37)	62.18 (5.37)	2.26 (0.23)
1 to 3	0.05	2.73 (0.16)	0.51 (0.03)	0.32 (0.07)	3.43 (0.22)	37.82 (5.37)	62.18 (5.37)	2.26 (0.23)
1 to 4	0.04	2.73 (0.16)	0.42 (0.03)	0.32 (0.07)	3.43 (0.22)	37.82 (5.37)	62.18 (5.37)	2.26 (0.23)
1 to 5	0.09	2.73 (0.16)	0.86 (0.05)	0.32 (0.07)	3.43 (0.22)	37.82 (5.37)	62.18 (5.37)	2.26 (0.23)
2 to 1	0.10	0.42 (0.07)	0.24 (0.04)	0.04 (0.01)	0.83 (0.16)	81.00 (5.92)	19.00 (5.92)	0.19 (0.06)
2 to 3	0.01	0.42 (0.07)	0.02 (0.003)	0.04 (0.01)	0.83 (0.16)	81.00 (5.92)	19.00 (5.92)	0.19 (0.06)
2 to 4	0.02	0.42 (0.07)	0.05 (0.01)	0.04 (0.01)	0.83 (0.16)	81.00 (5.92)	19.00 (5.92)	0.19 (0.06)
2 to 5	0.04	0.42 (0.07)	0.11 (0.02)	0.04 (0.01)	0.83 (0.16)	81.00 (5.92)	19.00 (5.92)	0.19 (0.06)
3 to 1	0.06	0.79 (0.13)	0.30 (0.05)	0.04 (0.03)	1.51 (0.29)	80.62 (4.99)	19.38 (4.99)	0.32 (0.10)
3 to 2	0.01	0.79 (0.13)	0.04 (0.01)	0.04 (0.03)	1.51 (0.29)	80.62 (4.99)	19.38 (4.99)	0.32 (0.10)
3 to 4	0.02	0.79 (0.13)	0.09 (0.01)	0.04 (0.03)	1.51 (0.29)	80.62 (4.99)	19.38 (4.99)	0.32 (0.10)
3 to 5	0.07	0.79 (0.13)	0.36 (0.06)	0.04 (0.03)	1.51 (0.29)	80.62 (4.99)	19.38 (4.99)	0.32 (0.10)
4 to 1	0.04	0.75 (0.08)	0.22 (0.02)	0.09 (0.02)	1.13 (0.12)	52.27 (5.96)	47.73 (5.96)	0.58 (0.09)
4 to 2	0.02	0.75 (0.08)	0.09 (0.01)	0.09 (0.02)	1.13 (0.12)	52.27 (5.96)	47.73 (5.96)	0.58 (0.09)
4 to 3	0.02	0.75 (0.08)	0.10 (0.01)	0.09 (0.02)	1.13 (0.12)	52.27 (5.96)	47.73 (5.96)	0.58 (0.09)
4 to 5	0.07	0.75 (0.08)	0.35 (0.04)	0.09 (0.02)	1.13 (0.12)	52.27 (5.96)	47.73 (5.96)	0.58 (0.09)
5 to 1	0.08	1.88 (0.11)	0.61 (0.04)	0.47 (0.16)	2.72 (0.36)	51.54 (9.29)	48.46 (9.29)	1.56 (0.31)
5 to 2	0.04	1.88 (0.11)	0.31 (0.02)	0.47 (0.16)	2.72 (0.36)	51.54 (9.29)	48.46 (9.29)	1.56 (0.31)
5 to 3	0.06	1.88 (0.11)	0.46 (0.03)	0.47 (0.16)	2.72 (0.36)	51.54 (9.29)	48.46 (9.29)	1.56 (0.31)
5 to 4	0.07	1.88 (0.11)	0.50 (0.03)	0.47 (0.16)	2.72 (0.36)	51.54 (9.29)	48.46 (9.29)	1.56 (0.31)

Cumulative dwell time (CDT) histograms were provided after TDP clustering in MASH-FRET. Corrected dwell times ($\tau_{jj'}$) were determined from values extracted from single-exponential fitting (τ_j) of the CDT histograms, by multiplying by the relative weight of the individual 2-D Gaussian in the mixture used to fit the TDP. For single-exponential and biexponential decay analysis, the error in τ (parentheses) was determined by BOBA-FRET analysis (4).

Supplementary table 6: State-to-state kinetics for mobile $(CAG)_{11}$ 3WJ with and without a CAA interrupt (see notes in Supp. Table 5)

$(CAG)_{11}$								
Transition	Relative	τ_j (s)	τ_{jj} (s)	τ (bi)1 (s)	τ (bi)2 (s)	A1 (%)	A2 (%)	τ (bi)av (s)
1 to 2	0.02	1.06 (0.08)	0.11 (0.01)	0.16 (0.05)	1.76 (0.43)	73.50 (8.14)	26.50 (8.14)	0.58 (0.19)
1 to 3	0.04	1.06 (0.08)	0.21 (0.02)	0.16 (0.05)	1.76 (0.43)	73.50 (8.14)	26.50 (8.14)	0.58 (0.19)
1 to 4	0.04	1.06 (0.08)	0.18 (0.01)	0.16 (0.05)	1.76 (0.43)	73.50 (8.14)	26.50 (8.14)	0.58 (0.19)
1 to 5	0.02	1.06 (0.08)	0.12 (0.01)	0.16 (0.05)	1.76 (0.43)	73.50 (8.14)	26.50 (8.14)	0.58 (0.19)
1 to 6	0.02	1.06 (0.08)	0.08 (0.01)	0.16 (0.05)	1.76 (0.43)	73.50 (8.14)	26.50 (8.14)	0.58 (0.19)
1 to 7	0.02	1.06 (0.08)	0.11 (0.01)	0.16 (0.05)	1.76 (0.43)	73.50 (8.14)	26.50 (8.14)	0.58 (0.19)
1 to 8	0.05	1.06 (0.08)	0.25 (0.02)	0.16 (0.05)	1.76 (0.43)	73.50 (8.14)	26.50 (8.14)	0.58 (0.19)
2 to 1	0.02	1.35 (0.14)	0.15 (0.02)	0.43 (0.07)	2.23 (0.30)	66.44 (7.79)	33.56 (7.79)	1.0 (0.21)
2 to 3	0.01	1.35 (0.14)	0.09 (0.01)	0.43 (0.07)	2.23 (0.30)	66.44 (7.79)	33.56 (7.79)	1.0 (0.21)
2 to 4	0.02	1.35 (0.14)	0.14 (0.01)	0.43 (0.07)	2.23 (0.30)	66.44 (7.79)	33.56 (7.79)	1.0 (0.21)
2 to 5	0.03	1.35 (0.14)	0.21 (0.02)	0.43 (0.07)	2.23 (0.30)	66.44 (7.79)	33.56 (7.79)	1.0 (0.21)
2 to 6	0.04	1.35 (0.14)	0.33 (0.03)	0.43 (0.07)	2.23 (0.30)	66.44 (7.79)	33.56 (7.79)	1.0 (0.21)
2 to 7	0.03	1.35 (0.14)	0.29 (0.03)	0.43 (0.07)	2.23 (0.30)	66.44 (7.79)	33.56 (7.79)	1.0 (0.21)
2 to 8	0.02	1.35 (0.14)	0.14 (0.01)	0.43 (0.07)	2.23 (0.30)	66.44 (7.79)	33.56 (7.79)	1.0 (0.21)
3 to 1	0.04	0.79 (0.12)	0.25 (0.04)	0.16 (0.05)	1.09 (0.14)	52.17 (5.80)	47.83 (5.80)	0.61 (0.09)
3 to 2	0.01	0.79 (0.12)	0.05 (0.01)	0.16 (0.05)	1.09 (0.14)	52.17 (5.80)	47.83 (5.80)	0.61 (0.09)
3 to 4	0.002	0.79 (0.12)	0.01 (0.002)	0.16 (0.05)	1.09 (0.14)	52.17 (5.80)	47.83 (5.80)	0.61 (0.09)
3 to 5	0.01	0.79 (0.12)	0.05 (0.01)	0.16 (0.05)	1.09 (0.14)	52.17 (5.80)	47.83 (5.80)	0.61 (0.09)
3 to 6	0.03	0.79 (0.12)	0.15 (0.02)	0.16 (0.05)	1.09 (0.14)	52.17 (5.80)	47.83 (5.80)	0.61 (0.09)
3 to 7	0.03	0.79 (0.12)	0.19 (0.03)	0.16 (0.05)	1.09 (0.14)	52.17 (5.80)	47.83 (5.80)	0.61 (0.09)
3 to 8	0.02	0.79 (0.12)	0.09 (0.01)	0.16 (0.05)	1.09 (0.14)	52.17 (5.80)	47.83 (5.80)	0.61 (0.09)
4 to 1	0.03	0.69 (0.18)	0.25 (0.06)	0.12 (0.03)	1.65 (0.72)	81.18 (12.91)	18.82 (12.91)	0.41 (0.25)
4 to 2	0.02	0.69 (0.18)	0.12 (0.03)	0.12 (0.03)	1.65 (0.72)	81.18 (12.91)	18.82 (12.91)	0.41 (0.25)
4 to 3	0.003	0.69 (0.18)	0.02 (0.01)	0.12 (0.03)	1.65 (0.72)	81.18 (12.91)	18.82 (12.91)	0.41 (0.25)
4 to 5	0.002	0.69 (0.18)	0.01 (0.003)	0.12 (0.03)	1.65 (0.72)	81.18 (12.91)	18.82 (12.91)	0.41 (0.25)
4 to 6	0.01	0.69 (0.18)	0.05 (0.01)	0.12 (0.03)	1.65 (0.72)	81.18 (12.91)	18.82 (12.91)	0.41 (0.25)
4 to 7	0.02	0.69 (0.18)	0.11 (0.03)	0.12 (0.03)	1.65 (0.72)	81.18 (12.91)	18.82 (12.91)	0.41 (0.25)
4 to 8	0.02	0.69 (0.18)	0.12 (0.03)	0.12 (0.03)	1.65 (0.72)	81.18 (12.91)	18.82 (12.91)	0.41 (0.25)
5 to 1	0.03	0.42 (0.05)	0.15 (0.02)	0.08 (0.01)	0.90 (0.19)	83.55 (4.52)	16.45 (4.52)	0.22 (0.05)
5 to 2	0.03	0.42 (0.05)	0.14 (0.02)	0.08 (0.01)	0.90 (0.19)	83.55 (4.52)	16.45 (4.52)	0.22 (0.05)
5 to 3	0.01	0.42 (0.05)	0.04 (0.01)	0.08 (0.01)	0.90 (0.19)	83.55 (4.52)	16.45 (4.52)	0.22 (0.05)
5 to 4	0.002	0.42 (0.05)	0.01 (0.001)	0.08 (0.01)	0.90 (0.19)	83.55 (4.52)	16.45 (4.52)	0.22 (0.05)
5 to 6	0.001	0.42 (0.05)	0.01 (0.001)	0.08 (0.01)	0.90 (0.19)	83.55 (4.52)	16.45 (4.52)	0.22 (0.05)
5 to 7	0.003	0.42 (0.05)	0.02 (0.002)	0.08 (0.01)	0.90 (0.19)	83.55 (4.52)	16.45 (4.52)	0.22 (0.05)
5 to 8	0.01	0.42 (0.05)	0.06 (0.01)	0.08 (0.01)	0.90 (0.19)	83.55 (4.52)	16.45 (4.52)	0.22 (0.05)
6 to 1	0.02	0.43 (0.05)	0.08 (0.01)	0.09 (0.02)	0.70 (0.17)	72.52 (9.37)	27.48 (9.37)	0.26 (0.08)
6 to 2	0.04	0.43 (0.05)	0.17 (0.02)	0.09 (0.02)	0.70 (0.17)	72.52 (9.37)	27.48 (9.37)	0.26 (0.08)
6 to 3	0.03	0.43 (0.05)	0.12 (0.01)	0.09 (0.02)	0.70 (0.17)	72.52 (9.37)	27.48 (9.37)	0.26 (0.08)
6 to 4	0.01	0.43 (0.05)	0.03 (0.003)	0.09 (0.02)	0.70 (0.17)	72.52 (9.37)	27.48 (9.37)	0.26 (0.08)
6 to 5	0.001	0.43 (0.05)	0.01 (0.001)	0.09 (0.02)	0.70 (0.17)	72.52 (9.37)	27.48 (9.37)	0.26 (0.08)
6 to 7	0.002	0.43 (0.05)	0.01 (0.001)	0.09 (0.02)	0.70 (0.17)	72.52 (9.37)	27.48 (9.37)	0.26 (0.08)
6 to 8	0.003	0.43 (0.05)	0.01 (0.001)	0.09 (0.02)	0.70 (0.17)	72.52 (9.37)	27.48 (9.37)	0.26 (0.08)
7 to 1	0.02	0.50 (0.06)	0.10 (0.01)	0.11 (0.02)	0.76 (0.21)	70.06 (9.26)	29.94 (9.26)	0.30 (0.10)
7 to 2	0.04	0.50 (0.06)	0.17 (0.02)	0.11 (0.02)	0.76 (0.21)	70.06 (9.26)	29.94 (9.26)	0.30 (0.10)
7 to 3	0.03	0.50 (0.06)	0.12 (0.02)	0.11 (0.02)	0.76 (0.21)	70.06 (9.26)	29.94 (9.26)	0.30 (0.10)
7 to 4	0.01	0.50 (0.06)	0.06 (0.01)	0.11 (0.02)	0.76 (0.21)	70.06 (9.26)	29.94 (9.26)	0.30 (0.10)
7 to 5	0.00	0.50 (0.06)	0.02 (0.002)	0.11 (0.02)	0.76 (0.21)	70.06 (9.26)	29.94 (9.26)	0.30 (0.10)
7 to 6	0.002	0.50 (0.06)	0.01 (0.001)	0.11 (0.02)	0.76 (0.21)	70.06 (9.26)	29.94 (9.26)	0.30 (0.10)
7 to 8	0.003	0.50 (0.06)	0.01 (0.001)	0.11 (0.02)	0.76 (0.21)	70.06 (9.26)	29.94 (9.26)	0.30 (0.10)
8 to 1	0.05	0.51 (0.05)	0.21 (0.02)	0.10 (0.02)	0.81 (0.21)	74.10 (9.31)	25.90 (9.31)	0.28 (0.10)
8 to 2	0.01	0.51 (0.05)	0.07 (0.01)	0.10 (0.02)	0.81 (0.21)	74.10 (9.31)	25.90 (9.31)	0.28 (0.10)
8 to 3	0.02	0.51 (0.05)	0.07 (0.01)	0.10 (0.02)	0.81 (0.21)	74.10 (9.31)	25.90 (9.31)	0.28 (0.10)
8 to 4	0.02	0.51 (0.05)	0.08 (0.01)	0.10 (0.02)	0.81 (0.21)	74.10 (9.31)	25.90 (9.31)	0.28 (0.10)
8 to 5	0.01	0.51 (0.05)	0.06 (0.01)	0.10 (0.02)	0.81 (0.21)	74.10 (9.31)	25.90 (9.31)	0.28 (0.10)
8 to 6	0.003	0.51 (0.05)	0.01 (0.001)	0.10 (0.02)	0.81 (0.21)	74.10 (9.31)	25.90 (9.31)	0.28 (0.10)
8 to 7	0.004	0.51 (0.05)	0.02 (0.002)	0.10 (0.02)	0.81 (0.21)	74.10 (9.31)	25.90 (9.31)	0.28 (0.10)
$(CAG)_5(CAA)(CAG)_5$								
Transition	Relative	τ_j (s)	τ_{jj} (s)	τ (bi)1 (s)	τ (bi)2 (s)	A1 (%)	A2 (%)	τ (bi)av (s)
1 to 2	0.07	2.21 (0.16)	0.76 (0.01)	0.21 (0.03)	4.91 (0.65)	81.65 (2.70)	18.35 (2.70)	1.07 (0.18)
1 to 3	0.06	2.21 (0.16)	0.67 (0.01)	0.21 (0.03)	4.91 (0.65)	81.65 (2.70)	18.35 (2.70)	1.07 (0.18)
1 to 4	0.03	2.21 (0.16)	0.33 (0.005)	0.21 (0.03)	4.91 (0.65)	81.65 (2.70)	18.35 (2.70)	1.07 (0.18)
1 to 5	0.04	2.21 (0.16)	0.45 (0.01)	0.21 (0.03)	4.91 (0.65)	81.65 (2.70)	18.35 (2.70)	1.07 (0.18)
2 to 1	0.07	1.66 (0.10)	0.62 (0.01)	0.27 (0.04)	2.34 (0.27)	61.59 (5.75)	38.41 (5.75)	1.07 (0.17)
2 to 3	0.05	1.66 (0.10)	0.45 (0.01)	0.27 (0.04)	2.34 (0.27)	61.59 (5.75)	38.41 (5.75)	1.07 (0.17)
2 to 4	0.04	1.66 (0.10)	0.37 (0.01)	0.27 (0.04)	2.34 (0.27)	61.59 (5.75)	38.41 (5.75)	1.07 (0.17)
2 to 5	0.08	1.66 (0.10)	0.77 (0.01)	0.27 (0.04)	2.34 (0.27)	61.59 (5.75)	38.41 (5.75)	1.07 (0.17)
3 to 1	0.06	1.14 (0.08)	0.61 (0.02)	0.17 (0.03)	1.92 (0.22)	74.44 (4.11)	25.56 (4.11)	0.62 (0.10)
3 to 2	0.05	1.14 (0.08)	0.50 (0.01)	0.17 (0.03)	1.92 (0.22)	74.44 (4.11)	25.56 (4.11)	0.62 (0.10)
3 to 4	0.02	1.14 (0.08)	0.20 (0.01)	0.17 (0.03)	1.92 (0.22)	74.44 (4.11)	25.56 (4.11)	0.62 (0.10)
3 to 5	0.09	1.14 (0.08)	0.89 (0.02)	0.17 (0.03)	1.92 (0.22)	74.44 (4.11)	25.56 (4.11)	0.62 (0.10)
4 to 1	0.03	0.57 (0.06)	0.51 (0.04)	0.11 (0.03)	0.85 (0.15)	67.57 (6.68)	32.43 (6.68)	0.35 (0.08)
4 to 2	0.04	0.57 (0.06)	0.72 (0.06)	0.11 (0.03)	0.85 (0.15)	67.57 (6.68)	32.43 (6.68)	0.35 (0.08)
4 to 3	0.02	0.57 (0.06)	0.40 (0.03)	0.11 (0.03)	0.85 (0.15)	67.57 (6.68)	32.43 (6.68)	0.35 (0.08)
4 to 5	0.03	0.57 (0.06)	0.57 (0.05)	0.11 (0.03)	0.85 (0.15)	67.57 (6.68)	32.43 (6.68)	0.35 (0.08)
5 to 1	0.04	0.87 (0.09)	0.39 (0.04)	0.19 (0.02)	1.44 (0.17)	73.39 (4.56)	26.61 (5.56)	0.52 (0.08)
5 to 2	0.08	0.87 (0.09)	0.72 (0.04)	0.19 (0.02)	1.44 (0.17)	73.39 (4.56)	26.61 (5.56)	0.52 (0.08)

Supplementary Figures

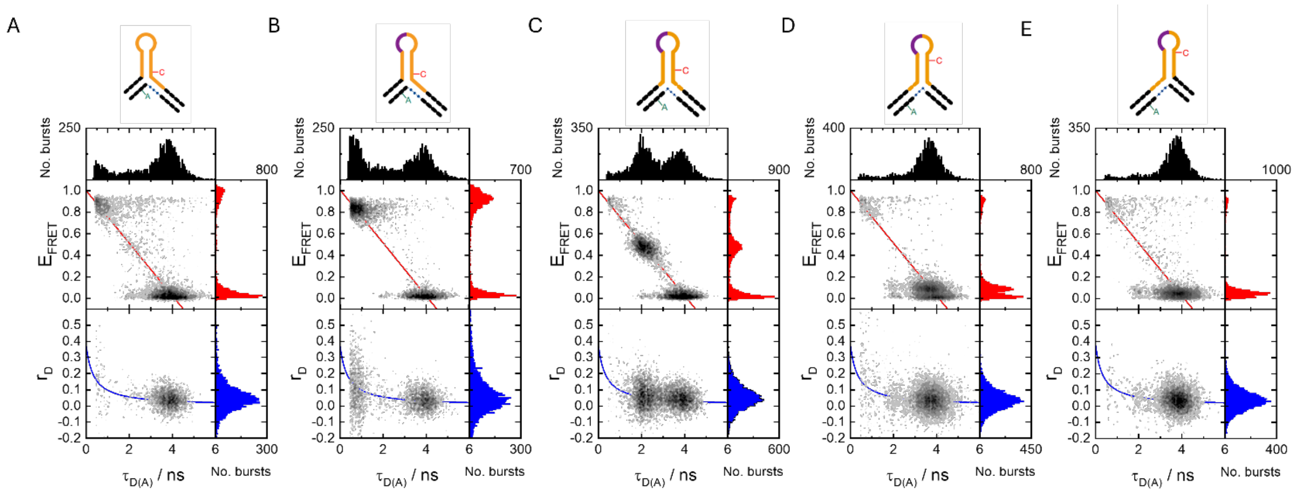

Supplementary figure 1. Confocal smFRET of mobile $(CAG)_{10}$ 3WJs with and without CAA interrupt. 2D MFD plot shows FRET efficiency (E_{FRET}) or donor anisotropy (r_D) vs donor lifetime [$\tau_{D(A)}$]. A) Freely diffusing $(CAG)_{10}$, B) CAA-1 C) CAA-2, D) CAA-3, E) CAA-4. The overlaid red line is the theoretical FRET relationship $E = 1 - [\tau_{D(A)}/\tau_D]$, where τ_D = 4.1 ns. The blue overlaid line is the Perrin equation $r_D = r_0/[1 + \tau_{D(A)}/\rho_D]$, with mean rotational correlation time ρ_D = 0.35 ns and fundamental anisotropy r_0 = 0.375. The gray scale indicates an increasing number of single-molecule bursts from white to black. The samples were measured at 20 ℃° in a buffer with 1 mM $MgCl_2$.

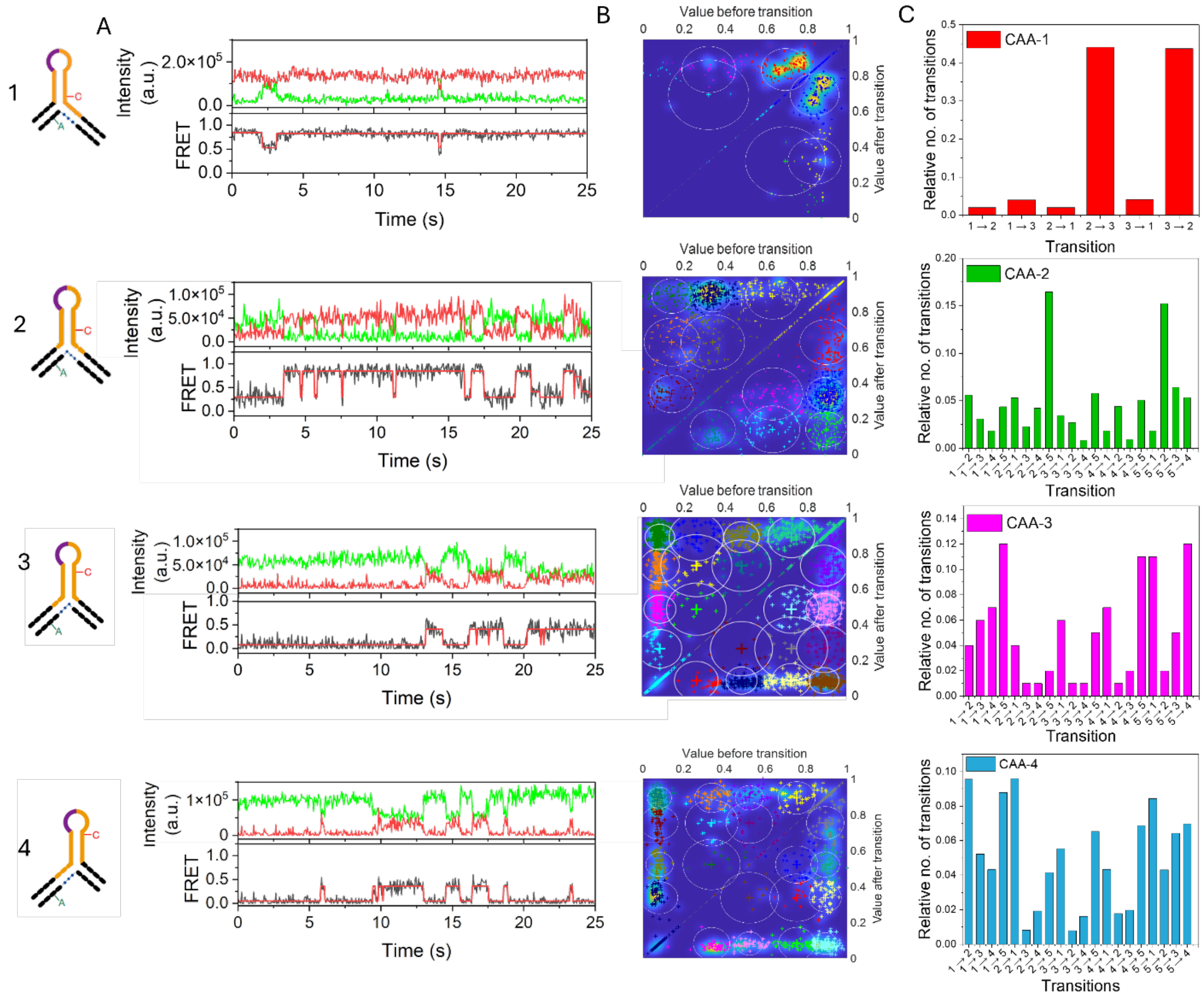

Supplementary figure 2. TIRF of mobile $(CAG)_{10}$ 3WJs containing CAA interrupts. Top to bottom (1-4) panels show analysis of immobilised samples CAA-1, CAA-2, CAA-3, and CAA-4, respectively. The samples were measured at 20 ℃ in a buffer with 1 mM $MgCl_2$. a) Exemplary TIRF time traces showing donor (green) and acceptor (red) signal (top) with corresponding FRET efficiency E_{FRET} (bottom) and HMM modelling (red line) (bottom). b) A clustering algorithm is applied to transition density plots in MASH-FRET to determine the overall state configuration. The most sufficient cluster model is determined by the Bayesian Information Criterion (BIC), which has the lowest value when the number of states (J) = 3, (J) = 5, (J) = 5 and (J) = 5 for samples 1-4 respectively. c) Transition density graphs showing the relative number of transitions to and from each FRET state.

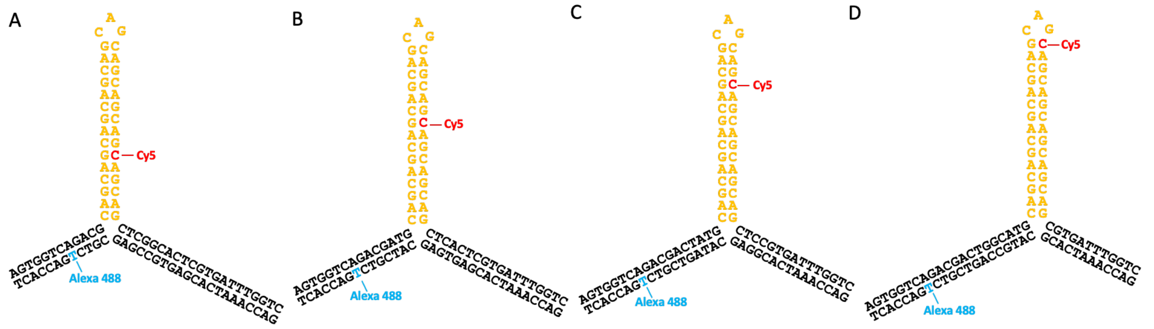

Supplementary figure 3. Structures of $(CAG)_{11}$ static 3WJs. A. S-CAG_{11}-1 **B.** S-CAG_{11}-2 **C.** S-CAG_{11}-3 **D.** S-CAG_{11}-4.

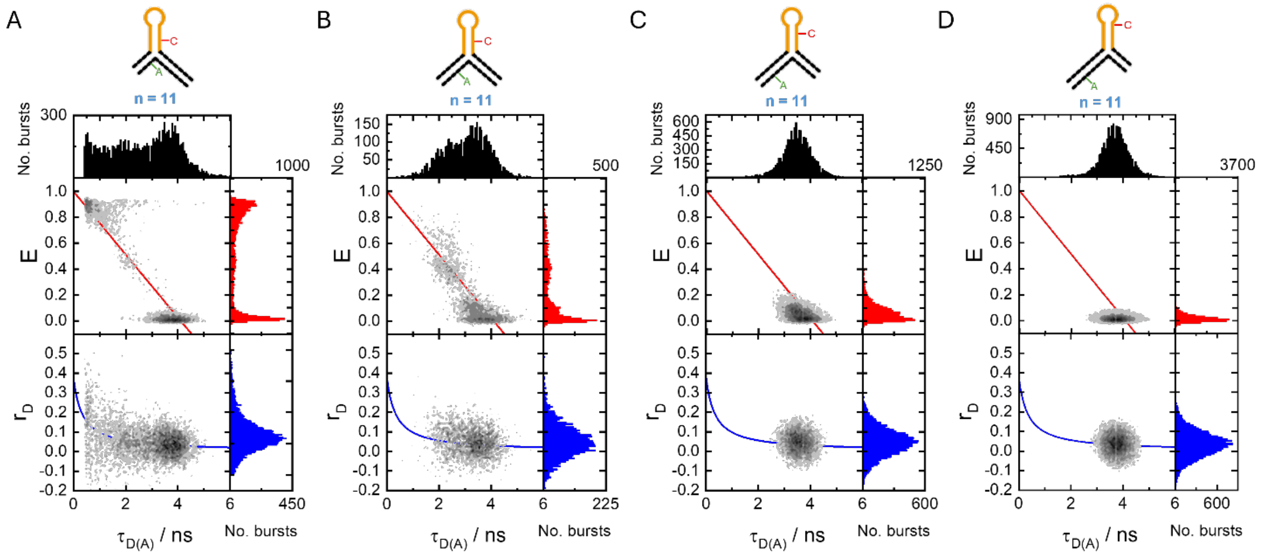

Supplementary figure 4. Confocal smFRET of static (CAG)$_{11}$ 3WJs. 2D MFD plot shows FRET efficiency (E_{FRET}) or donor anisotropy (r_D) vs donor lifetime [$\tau_{D(A)}$] of freely diffusing static 3WJs with an odd number of CAG repeats: A) S-CAG$_{11}$-1, B) S-CAG$_{11}$-2, C) S-CAG$_{11}$-3 and D) S-CAG$_{11}$-4.The overlaid red line is the theoretical FRET relationship $E = 1 - [\tau_{D(A)}/\tau_D]$, where τ_D = 4.1 ns. The blue overlaid line is the Perrin equation $r_D = r_0/[1 + \tau_{D(A)}/\rho_D]$, with mean rotational correlation time ρ_D = 0.35 ns and fundamental anisotropy r_0 = 0.375. The gray scale indicates an increasing number of single-molecule bursts from white to black. The samples were measured at 20 ℃ in a buffer with 1 mM $MgCl_2$.

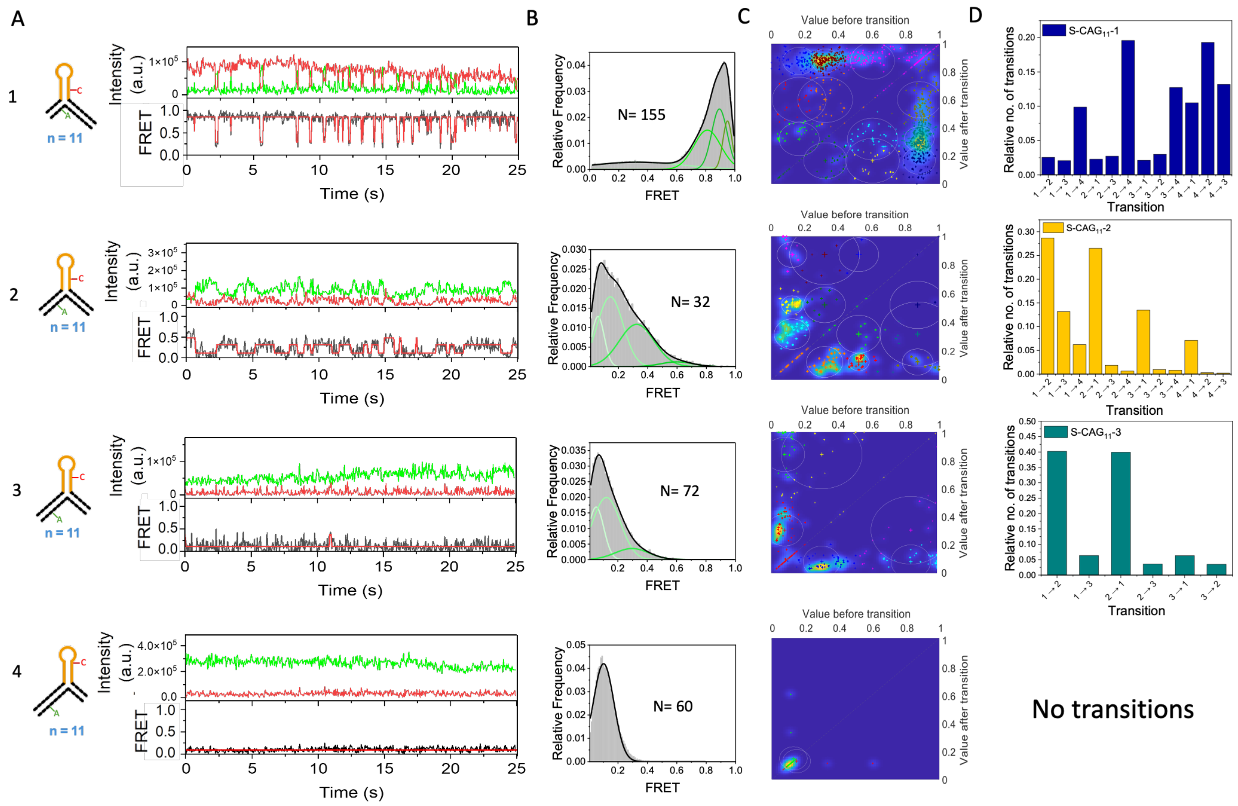

Supplementary figure 5. TIRF of static (CAG)$_{11}$ 3WJs. Top to bottom (1-4) panels show analysis of immobilised samples S-CAG$_{11}$-1, S-CAG$_{11}$-2, S-CAG$_{11}$-3 and S-CAG$_{11}$-4, respectively. The samples were measured at 20 ℃ in a buffer with 1 mM $MgCl_2$. A) Exemplary TIRF time traces showing donor (green) and acceptor (red) signal (top) with corresponding FRET efficiency E_{FRET} (bottom) and HMM modelling (red line) (bottom). B) FRET histograms from TIRF. The number of single molecules (N) measured is indicated in each panel. C) A clustering algorithm is applied to transition density plots in MASH-FRET to determine the overall state configuration. The most sufficient cluster model is determined by the Bayesian Information Criterion (BIC), which has the lowest value when the number of states (J) = 4, (J) = 4, (J) = 3 and (J) = 1 for samples 1-4, respectively. D) Transition density graphs showing the relative number of transitions to and from each FRET state (not shown for S-CAG$_{11}$-4 due to the existence of one state only).

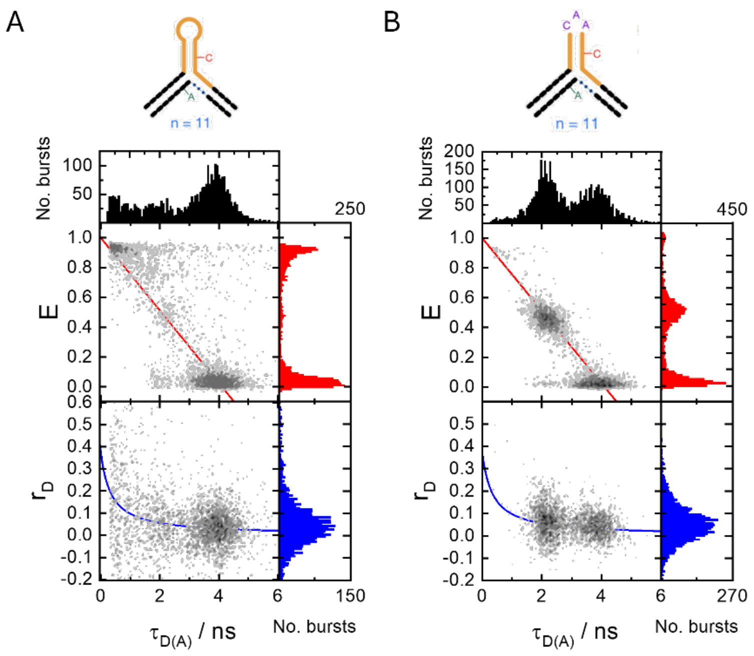

Supplementary figure 6. Confocal smFRET of mobile (CAG)$_{11}$ 3WJs with and without CAA interrupt. 2D MFD plot shows FRET efficiency (E_{FRET}) or donor anisotropy (r_D) vs donor lifetime [$\tau_{D(A)}$] for A) Freely diffusing (CAG)$_{11}$ and B) (CAG)$_5$(CAA)(CAG)$_5$. The overlaid red line is the theoretical FRET relationship $E = 1 - [\tau_{D(A)}/\tau_D]$, where τ_D = 4.1 ns. The blue overlaid line is the Perrin equation $r_D = r_0/[1 + \tau_{D(A)}/\rho_D]$, with mean rotational correlation time ρ_D = 0.35 ns and fundamental anisotropy r_0 = 0.375. The gray scale indicates an increasing number of single-molecule bursts from white to black. The samples were measured at 20 ℃ in a buffer with 1 mM $MgCl_2$.

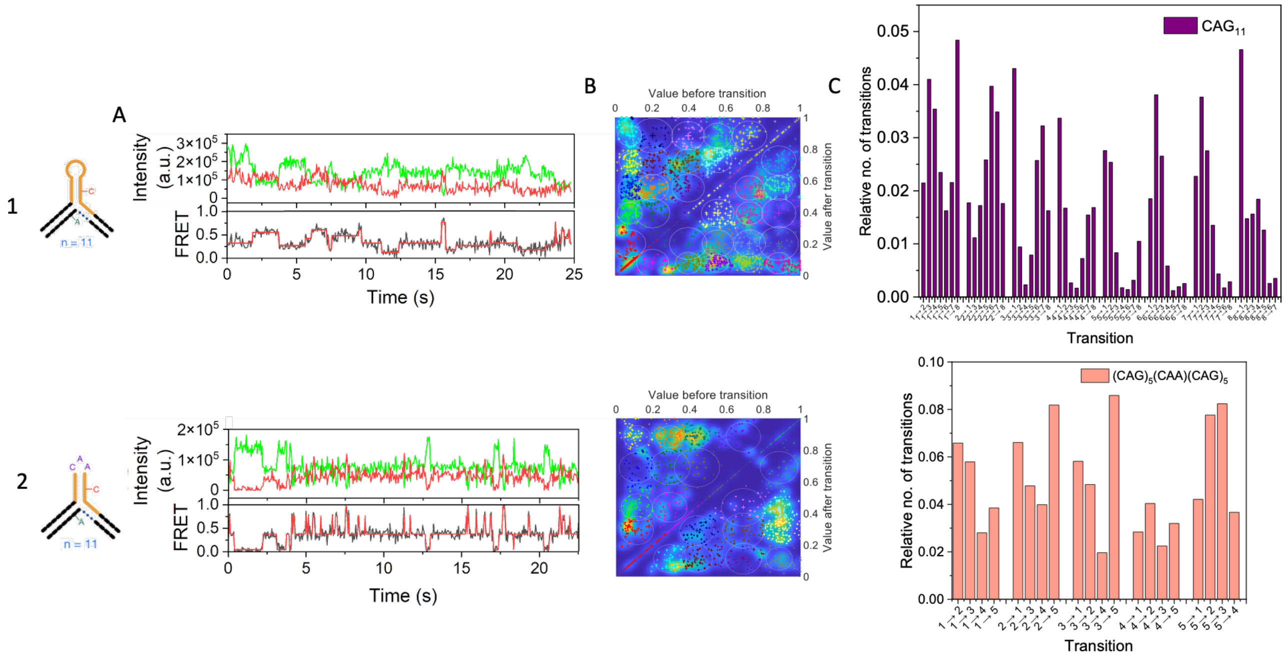

Supplementary figure 7. TIRF of mobile (CAG)$_{11}$ 3WJs with and without CAA interrupts. Analysis of immobilised samples (CAG)$_{11}$ (top) and (CAG)$_5$(CAA)(CAG)$_5$ (bottom). The samples were measured at 20 ℃ in a buffer with 1 mM $MgCl_2$. a) Exemplary TIRF time traces showing donor (green) and acceptor (red) signal (top) with corresponding FRET efficiency E_{FRET} (bottom) and HMM modelling (red line) (bottom). b) A clustering algorithm is applied to transition density plots in MASH-FRET to determine the overall state configuration. The most sufficient cluster model is determined by the Bayesian Information Criterion (BIC), which has the lowest value when the number of states (J) = 8, (J) = 5, 1-2, respectively. c) Transition density graphs showing the relative number of transitions to and from each FRET state.

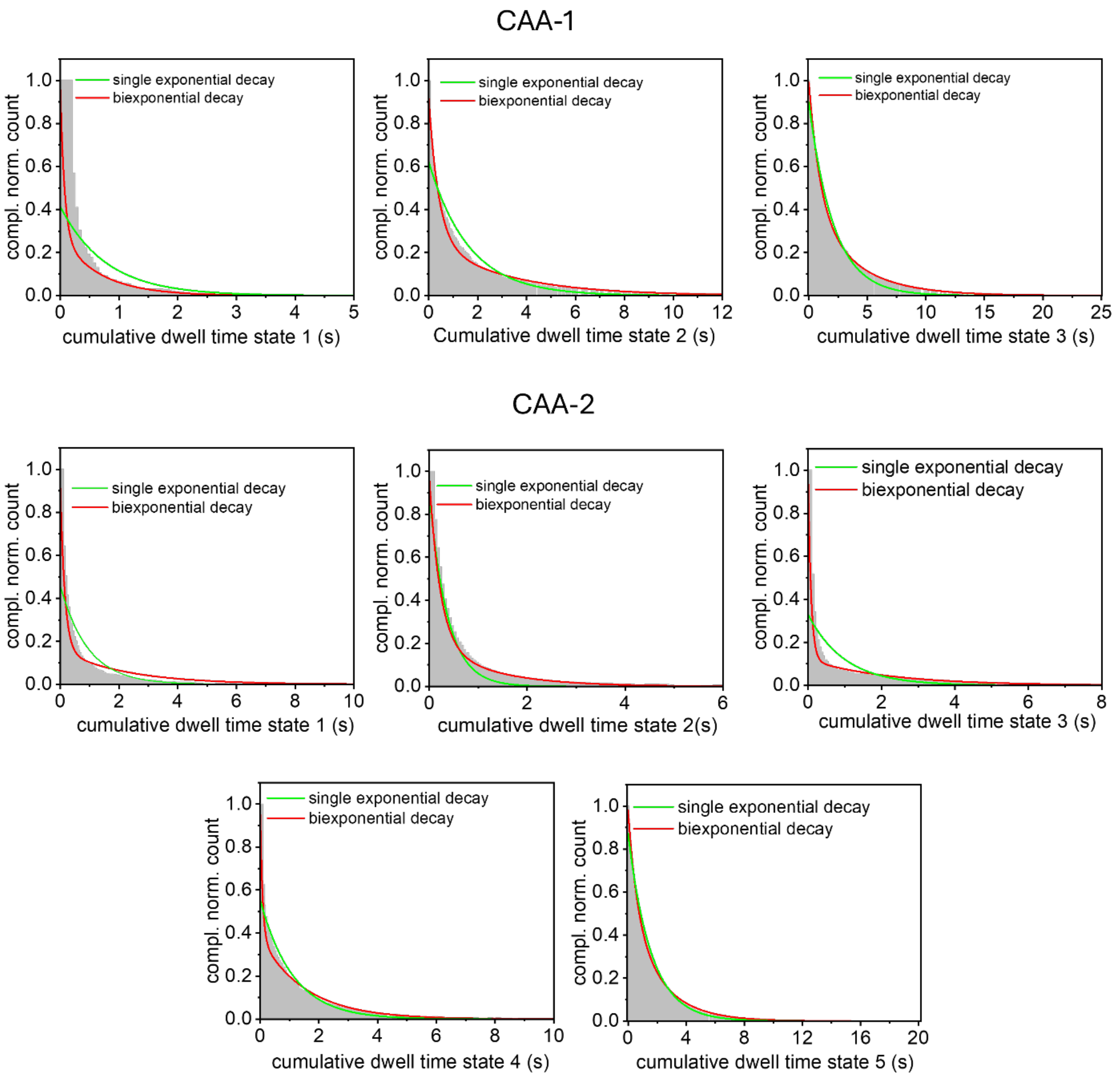

Supplementary figure 8. Cumulative frequency plots for CAA-1 and CAA-2. Dwell time histograms (shown in grey) were fitted to single exponential (green) and biexponential (red) decay functions in MASH-FRET and dwell times were computed from the fit.

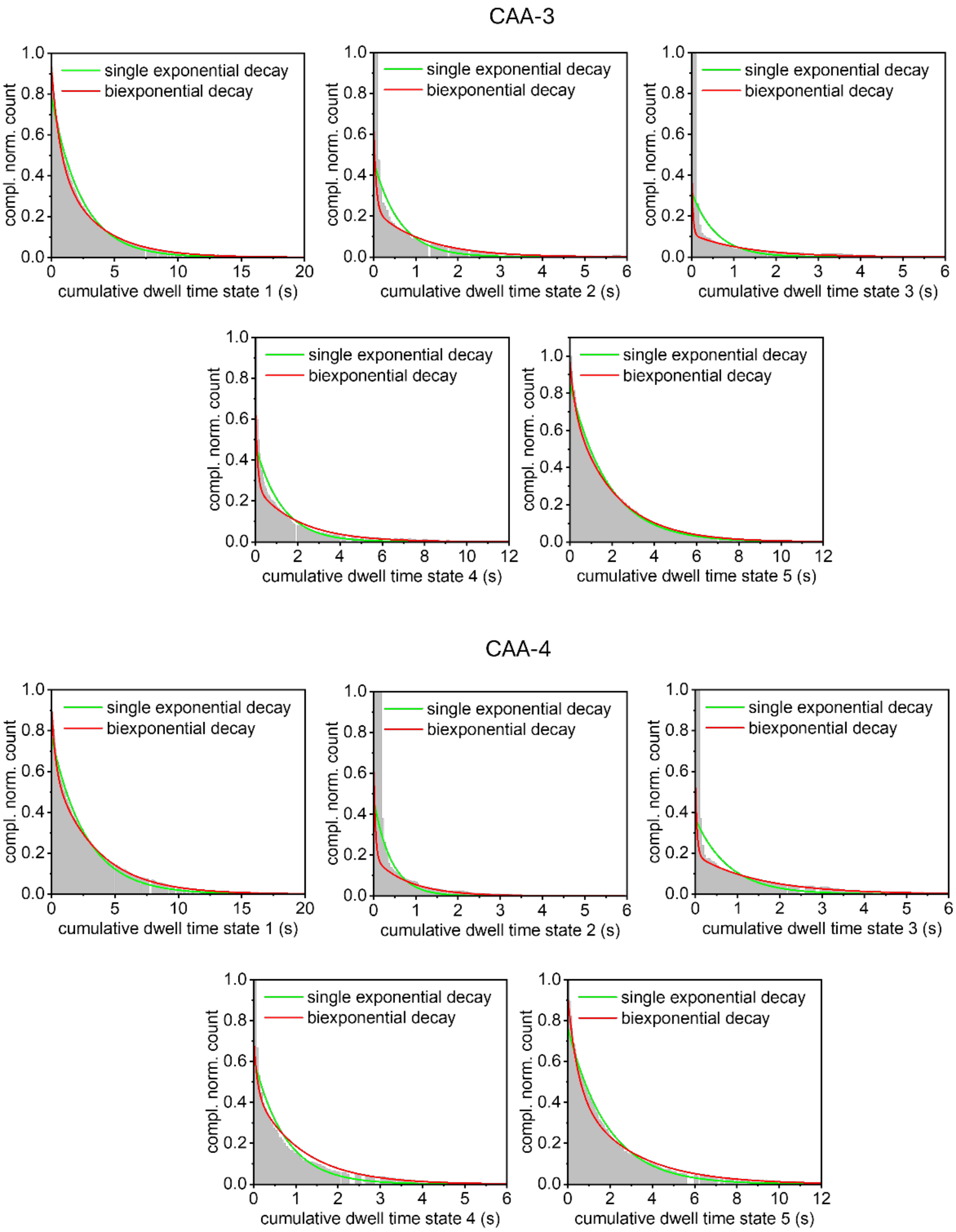

Supplementary figure 9. Cumulative frequency plots for CAA-3 and CAA-4. Dwell time histograms (shown in grey) were fitted to single exponential (green) and biexponential (red) decay functions in MASH-FRET and dwell times were computed from the fit.

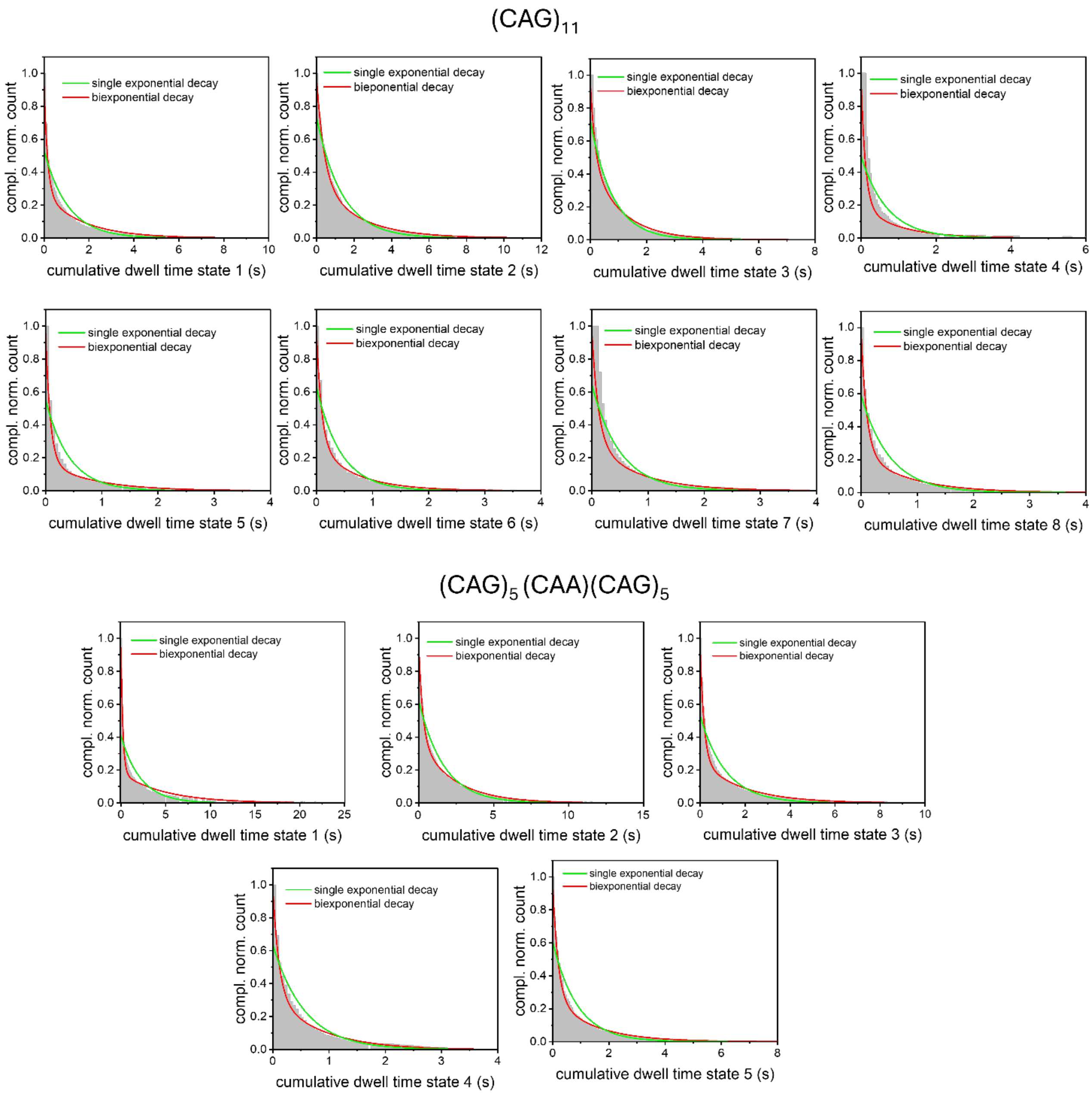

Supplementary figure 10. Cumulative frequency plots for $(CAG)_{11}$ and $(CAG)_5(CAA)(CAG)_5$. Dwell time histograms (shown in grey) were fitted to single exponential (green) and biexponential (red) decay functions in MASH-FRET and dwell times were computed from the fit.

References

1. Hu, T., Morten, M.J. and Magennis, S.W. (2021) Conformational and migrational dynamics of slipped-strand DNA three-way junctions containing trinucleotide repeats. *Nat. Commun.*, **12**, 204.
2. Bianco, S., Hu, T., Henrich, O. and Magennis, S.W. (2022) Heterogeneous migration routes of DNA triplet repeat slip-outs. *Biophysical Reports*, **2**, 100070.
3. Hadzic, M.C.A.S., Boerner, R., Koenig, S.L.B., Kowerko, D. and Sigel, R.K.O. (2018) Reliable state identification and state transition detection in fluorescence intensity-based single-molecule Förster resonance energy-transfer data. *J. Phys. Chem. B*, **122**, 6134-6147.
4. Koenig, S.L.B., Hadzic, M., Fiorini, E., Boerner, R., Kowerko, D., Blanckenhorn, W.U. and Sigel, R.K.O. (2013) BOBA FRET: bootstrap-based analysis of single-molecule FRET data. *PLoS One*, **8** (12), e84157.
